# Molecular identification of bulbospinal ON neurons by GPER, which drives pain and morphine tolerance

**DOI:** 10.1172/JCI154588

**Published:** 2023-01-03

**Authors:** Yingfu Jiao, Po Gao, Li Dong, Xiaowei Ding, Youqiang Meng, Jiahong Qian, Ting Gao, Ruoxi Wang, Tao Jiang, Yunchun Zhang, Dexu Kong, Yi Wu, Sihan Chen, Saihong Xu, Dan Tang, Ping Luo, Meimei Wu, Li Meng, Daxiang Wen, Changhao Wu, Guohua Zhang, Xueyin Shi, Weifeng Yu, Weifang Rong

**Affiliations:** 1 Department of Anatomy and Physiology and; 2 Department of Anesthesiology, Renji Hospital, Shanghai Jiao Tong University School of Medicine, Shanghai, China.; 3 Department of Neurosurgery, Xinhua Hospital, Shanghai Jiao Tong University School of Medicine, Chongming Branch, Shanghai University of Medicine and Health Sciences Affiliated Chongming Hospital, Shanghai, China.; 4 School of Biosciences and Medicine, University of Surrey, Guildford, United Kingdom.; 5 Department of Anesthesiology, Xinhua Hospital, Shanghai Jiao Tong University School of Medicine, Shanghai, China.

**Keywords:** Neuroscience, Pain

## Abstract

The rostral ventromedial medulla (RVM) exerts bidirectional descending modulation of pain attributable to the activity of electrophysiologically identified pronociceptive ON and antinociceptive OFF neurons. Here, we report that GABAergic ON neurons specifically express G protein–coupled estrogen receptor (GPER). GPER^+^ neurons exhibited characteristic ON-like responses upon peripheral nociceptive stimulation. Optogenetic activation of GPER^+^ neurons facilitated, but their ablation abrogated, pain. Furthermore, activation of GPER caused depolarization of ON cells, potentiated pain, and ameliorated morphine analgesia through desensitizing μ-type opioid receptor–mediated (MOR-mediated) activation of potassium currents. In contrast, genetic ablation or pharmacological blockade of GPER attenuated pain, enhanced morphine analgesia, and delayed the development of morphine tolerance in diverse preclinical pain models. Our data strongly indicate that GPER is a marker for GABAergic ON cells and illuminate the mechanisms underlying hormonal regulation of pain and analgesia, thus highlighting GPER as a promising target for the treatment of pain and opioid tolerance.

## Graphical abstract

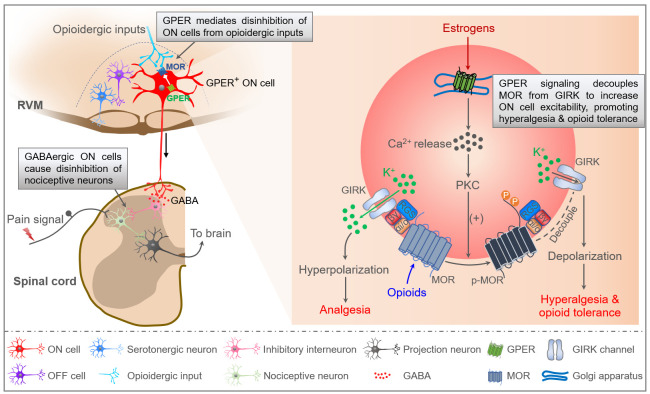

## Introduction

It is well known that the brain may impose powerful bidirectional descending control on the ascending transmission of pain signals and that pain sensation is ultimately determined by integrated ascending transmission and descending modulation (1–3). The neural substrate mediating descending pain modulation is complex and comprises multiple interactive pathways, among which the periaqueductal gray–rostral ventromedial medulla (PAG-RVM) system has been the most extensively studied and arguably the most important pain modulation pathway (4, 5). Electrical stimulation of the PAG induces analgesia and inhibition of nociceptive neurons in the dorsal horn (6). The analgesic influence of the PAG is mediated through extensive projections to the RVM, which consists of the nucleus raphe magnus and the adjacent reticular formation (2, 7, 8). Stimulation of the RVM may induce either analgesia or hyperalgesia, depending on the stimulation intensity and types of pain tested (9). Such bidirectional effects have been attributed to the activity of 2 subsets of spinally projecting neurons in the RVM, ON cells and OFF cells, which are presumed to facilitate and inhibit ascending pain transmission, respectively (10, 11). The RVM is also an important site for the analgesic effects of endogenous or exogenous opioids, since opioid analgesia is impaired following ablation of the RVM (7, 12).

Considerable preclinical evidence suggests that an imbalance between descending facilitation and descending inhibition, particularly heightened descending facilitation from the RVM, may underlie central sensitization in various pathological pain conditions (13–15). This conclusion has been reinforced by recent neuroimaging studies in humans, which demonstrated altered functional connectivity of the RVM in chronic pain conditions (13–16). However, significant controversies and ambiguities exist in the field. For example, the specific role of the PAG-RVM in pain regulation has been challenged, since these structures are also involved in the regulation of physiological functions such as micturition, thermoregulation, and motor control for the maintenance of homeostasis (17, 18). The intrinsic cellular organization of the RVM is still poorly defined. ON and OFF cells are believed to facilitate and inhibit ascending nociceptive transmission, respectively, but it remains a major challenge to selectively identify, manipulate, or monitor the activity of ON and OFF cells, given the lack of specific markers (19).

On the other hand, the female hormone estrogens have long been suspected to play significant roles in pain regulation, given the well-documented sexual dimorphisms of pain and analgesia (20–22). However, few studies have directly investigated the estrogenic effects on the descending pain modulation systems. We postulated that estrogens may affect pain and analgesia via alteration of neuronal activities in the RVM and attempted to delineate the cellular and receptor mechanisms that may underlie the estrogenic actions. We found that estrogen delivered to the RVM led to rapid potentiation of pain and amelioration of morphine analgesia. Surprisingly, the receptor mediating such effects, G protein–coupled estrogen receptor (GPER), was found to specifically identify a population of GABAergic ON cells. We found that GPER plays a major role in the control of ON cell excitability through modulation of μ-opioid signaling, which may contribute to chronic pain and morphine tolerance.

## Results

### Estrogen in the RVM facilitates pain and ameliorates morphine analgesia through activation of GPER.

**Figure 1:**
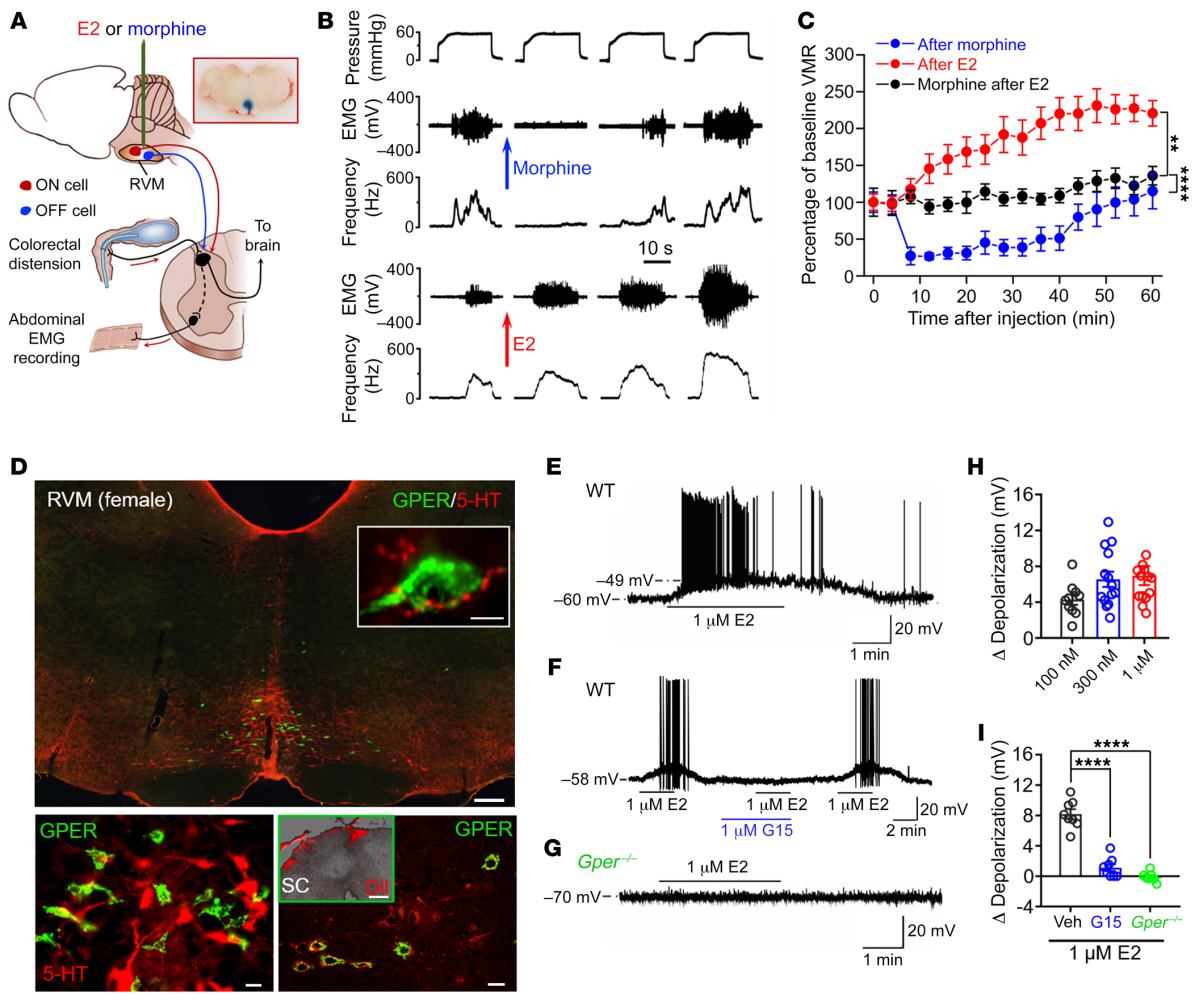
Estrogen facilitates pain and ameliorates morphine analgesia through activation of GPER. (**A**) Schematic showing the experimental model in which the influence of E2 on the descending pain modulation system was tested. The image at the upper right corner shows a typical injection site labeled through microinjection of 1% pontamine sky blue. (**B**) Sequential recordings showing morphine inhibited, whereas E2 potentiated, the VMR. (**C**) Relative change in CRD-induced VMR responses following local administration of morphine or E2 to the RVM, with the black line showing that morphine analgesia was negated 15 minutes after intracisternal application of E2. *n* = 6 rats/group. (**D**) Double immunofluorescence showing distinctive GPER expression in the RVM in adult female rats. Note the lower left panel, which shows that GPER expression was exclusively confined to nonserotonergic neurons. The inset in the upper panel shows a 5-HT^+^ terminal fiber in close proximity to a GPER^+^ soma. The lower right panel shows GPER expression in DiI-labeled neurons, with the inset showing DiI fluorescence on the dorsal surface of the thoracic spinal cord (SC) where DiI was applied. Scale bars: 200 μm (upper panel), 10 μm (inset in upper panel, 20 μm (lower panels), and 300 μm (inset in lower right panel). (**E**) Brain slice electrophysiology showing that E2 caused rapid depolarization of RVM neurons in WT female rats. (**F** and **G**) E2-induced membrane depolarization was absent in the presence of G15 and in *Gper^–/–^* rats. (**H**) Average amplitude of membrane depolarization induced by different concentrations of E2. *n* = 10–14 cells/group. (**I**) Negating effects of G15 and *Gper* deficiency on E2-induced membrane depolarization. *n* = 7–8/group. Data are representative of at least 3 independent experiments and are presented as the mean ± SEM. **P < 0.01 and ****P < 0.0001, by 1-way ANOVA followed by Tukey’s post hoc test (**C**, **H**, and **I**).

To test the hypothesis that estrogens may affect the activity of the PAG-RVM system, we microinjected 17β-estradiol (E2) into the RVM and observed its effect on the visceromotor responses evoked by noxious colorectal distension (i.e., the colorectal distension–induced [CRD-induced] visceral motor response [VMR]) in isoflurane-anesthetized adult female rats (Figure 1A). We chose to study female rats, as estrogen signaling is generally considered to be more important in the female than in the male. As illustrated in Figure 1, B and C, morphine (2 μg in 0.5 μL) applied to the RVM caused a profound inhibition of the VMR, consistent with the notion that the RVM is an important site of morphine analgesia. In direct contrast, microinjection of E2 (0.1–1 μM, 0.5 L) into the RVM led to rapid and incremental increases in the VMR, and this effect was dose dependent (Figure 1, B and C, and Supplemental Figure 1A, which also indicates that microinjection of 0.01 μM E2 and the same volume of saline had no effect on the VMR). Furthermore, application of E2 (1 μM, 10 L) intracisternally negated the inhibitory effect of locally applied morphine on the VMR (Figure 1C). These data showed that E2 acting in the RVM might lead to facilitation of visceral nociception and amelioration of morphine analgesia.

Traditionally, estrogens are known to bind the nuclear receptors ERα and ERβ, which act as ligand-activated transcription factors to regulate gene transcription and protein synthesis. More recently, however, an orphan GPCR, namely GPER, has been demonstrated to mediate rapid estrogenic signaling (23–25). To delineate which receptor was responsible for the E2 effect in the RVM, we determined the expression of ERα, ERβ, and GPER by immunofluorescence. To our surprise, there was an abundance of GPER immunoreactivity distinctively localized in the RVM of female (Figure 1D) and male (Supplemental Figure 1C) rats, whereas ERα and ERβ were expressed at relatively low levels in the RVM (Supplemental Figure 1B; supplemental material available online with this article; https://doi.org/10.1172/JCI154588DS1). Serotonergic neurons constitute approximately 23% of the neuronal population in the RVM (26) and are known to be involved in descending modulation of pain (27), however, GPER immunoreactivity was exclusively confined to nonserotonergic neurons in the RVM, and it was found that GPER^+^ neurons were intermingled with serotonergic neurons in the RVM, with some varicose 5-hydroxytryptamine–positive (5-HT^+^) terminal fibers seen in close proximity to GPER^+^ soma (Figure 1D). Compared with the RVM, GPER immunoreactivity was barely detectable in the PAG or in the adjacent dorsal raphe nucleus (DRN) or medial raphe nucleus (MRN) (Supplemental Figure 1D). Furthermore, by double labeling with the retrograde tracer 1,1′-dilinoleyl-3,3,3′,3′-tetramethylindocarbocyanine perchlorate (DiI) preapplied to the T12-L1 superficial dorsal horn, we found that GPER^+^ neurons in the RVM were colabeled with DiI (Figure 1D). Coimmunostaining for GPER and GAD67 showed that GPER^+^ neurons were positive for GAD67 (Supplemental Figure 1E). These data suggested that GPER^+^ neurons in the RVM are spinally projecting GABAergic neurons.

We further analyzed the rostral-caudal distribution of GPER^+^ neurons in female and male rats by double-immunostaining of GPER and 5-HT (Supplemental Figure 2). We found that GPER^+^ neurons were confined to the RVM either in female and male rats, however, there appeared to be sex differences in the number and distribution of GPER^+^ neurons. In the female, GPER^+^ neurons were largely restricted to the raphe nuclei, whereas in the male, GPER^+^ neurons were also present in large numbers in the gigantocellular reticular nucleus, alpha and ventral parts (GiA and GiV).

To test whether GPER indeed mediates the estrogenic effects in the RVM, a GPER-selective agonist G-1 (10 μM, 0.5 L) was microinjected into the RVM and found to mimic the effects of E2, causing rapid potentiation of a CRD-induced VMR and attenuation of morphine analgesia (Supplemental Figure 3). Furthermore, we conducted current-clamp recordings in medullary slices using WT and *Gper*-knockout (*Gper^–/–^*) rats (28). In the WT slices, E2 induced a rapid membrane depolarization in 38 of 75 neurons in a concentration-dependent manner (Figure 1, E and H). We did not observe this effect in the presence of the GPER-selective antagonist G15 or in *Gper^–/–^* slices (Figure 1, F, G, and I). Additionally, the GPER agonist G-1 (1 μM) also caused depolarization in 14 of 25 RVM neurons in WT slices (Supplemental Figure 4, A and B), and E2-induced (1 μM) depolarization was not significantly affected by the presence of the ERα antagonist methyl-piperidino-pyrazole (MPP) or the ERβ antagonist 4-[2-phenyl-5,7-bis(trifluoromethyl)pyrazolo[1,5-a]pyrimidin-3-yl]phenol (PHTPP) (Supplemental Figure 4, C–F). These data suggested that the estrogenic effects on RVM neuronal activity were primarily mediated by GPER.

The above results revealed a previously unappreciated, distinct expression of GPER in the RVM, and activation of GPER may profoundly affect descending modulation of pain and morphine analgesia.

### ON cells exclusively express GPER in the RVM.

**Figure 2:**
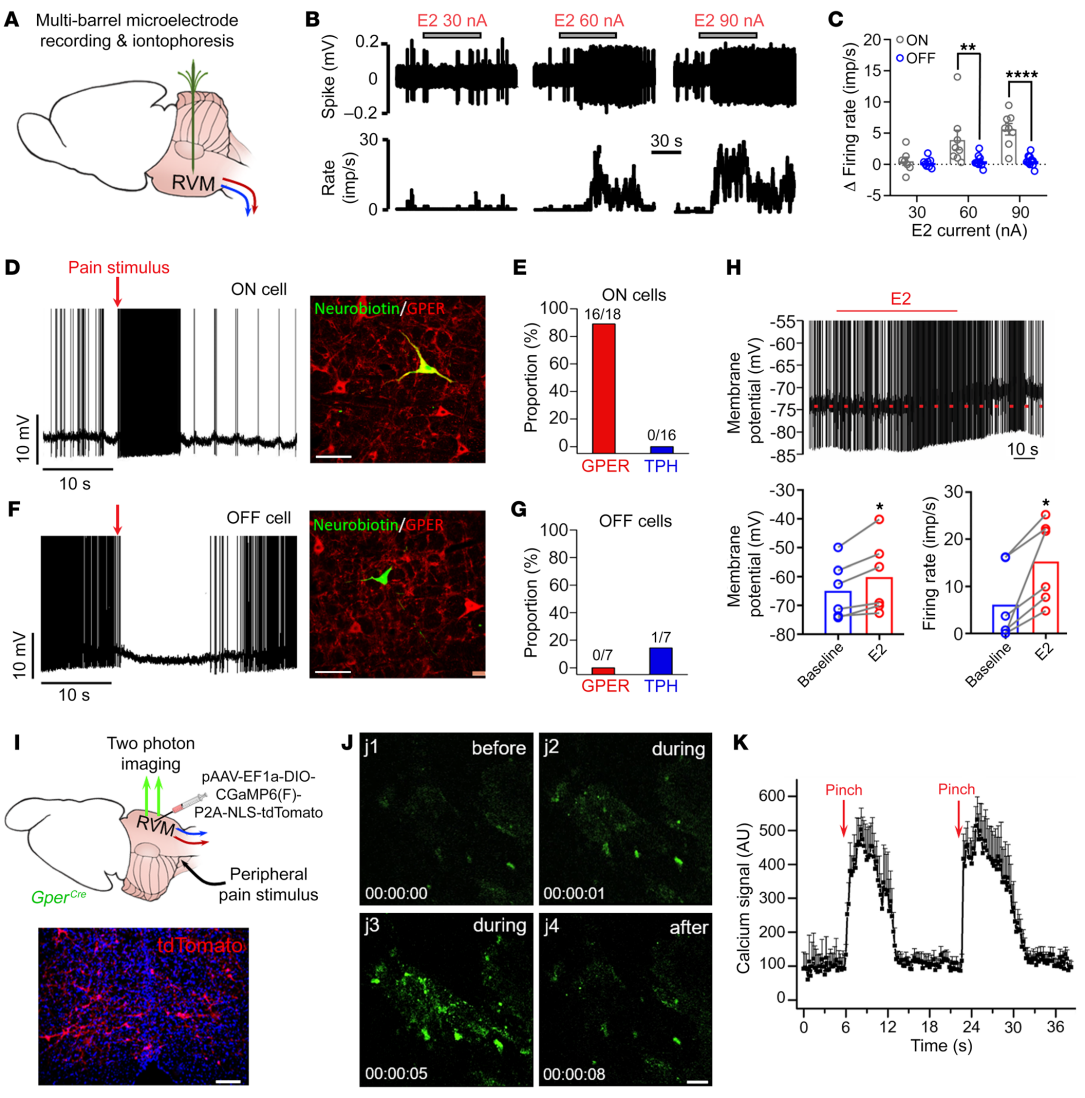
ON cells exclusively express GPER in the RVM. (**A**) Schematic of the experimental model. (**B** and **C**) Iontophoretic application of E2 caused dose-dependent (current-dependent) excitation of RVM ON cells without affecting OFF cells. *n* = 7–11 cells/group. (**D** and **E**) Intracellular recording, functional identification, and immunolabeling revealed positive GPER expression for the great majority (16 of 18) of RVM ON cells. The ON cells tested (*n* = 16) were not immunoreactive for TPH (see Supplemental Figure 4F for representative TPH immunofluorescence). Scale bar: 100 μm. (**F** and **G**) Intracellular recording, functional identification, and immunolabeling showing an absence of GPER immunoreactivity in RVM OFF neurons (*n* = 7). One of the 7 OFF neurons tested was immunoreactive to TPH. Scale bar: 40 μm. (**H**) ON cells recorded using the piggyback electrodes showed membrane potential depolarization and accelerated firing rates following iontophoretic application of E2. *n* = 6–7 cells/group. (**I**) Schematic showing in vivo 2-photon calcium imaging of RVM GPER^+^ neurons through surgery exposure from the ventral side in mice. *Gper^Cre^* mice were injected with pAAV-EF1a-DIO-CGaMP6(F)-P2A-NLS-tdTomato into the RVM to induce Cre-driven expression of GCaMP6 in GPER^+^ neurons. The image shows tdTomato^+^ fluorescence in RVM neurons infected with virus. Scale bar: 100 μm. (**J**) TdTomato^+^ (GPER^+^) neurons exhibited a transient rise in calcium in response to a noxious pinch applied to the hind paw. Scale bar: 100 μm. (**K**) Averaged calcium signal of 63 neurons from 5 mice during a noxious pinch applied to the hind paw. Data are representative of at least 3 independent experiments and are presented as the mean ± SEM. *P < 0.05, **P < 0.01, and ****P < 0.0001, by 2-way ANOVA followed by Bonferroni’s post hoc test (**C**) and paired, 2-tailed Student’s *t* test (**H**).

We wondered whether ON or OFF cells might express GPER and mediate the pronociceptive action of E2 in the RVM. To address this question, we carried out extracellular recording of RVM neurons using multibarrel electrodes to observe how E2 applied by iontophoresis would affect the activity of ON and OFF neurons in anesthetized rats (Figure 2A). ON and OFF cells were identified on the basis of their responses to peripheral noxious stimulation (29). Characteristically, ON cells exhibited excitatory responses, whereas OFF cells displayed inhibitory responses during noxious somatic (tail-flick test or hind-paw pinch) or visceral (colorectal distension to 60 mmHg) stimulation (Supplemental Figure 5A). The recording sites and spontaneous firing rates are shown in Supplemental Figure 5, B and C.

Application of E2 by iontophoresis rapidly increased the firing rate of ON cells without affecting the firing rate of OFF or neutral cells (Figure 2, B and C, and Supplemental Figure 5, D and E). To determine whether this ON cell–specific estrogenic effect was due to selective expression of GPER in ON cells, we performed intracellular recordings with single sharp electrodes filled with neurobiotin (1% in 3 M KCl). Following the functional identification of ON and OFF cells, each cell was labeled through iontophoretic injection of neurobiotin and subsequently coimmunostained for neurobiotin and GPER or tryptophan hydroxylase (TPH). Remarkably, the great majority (16 of 18) of ON cells tested were GPER immunoreactive (Figure 2, D and E), whereas none of the OFF neurons tested (*n* = 7) had GPER immunoreactivity (Figure 2, F and G). Consistent with the findings that GPER and 5-HT immunoreactivity was mutually exclusive (Figure 1), none of the ON cells tested (*n* = 16) showed TPH immunostaining (Figure 2E, and Supplemental Figure 5F). We then performed intracellular recordings with piggyback electrodes (a sharp recording electrode glued to multibarrel iontophoresis electrodes, Supplemental Figure 4G) to observe the effect of iontophoretically applied morphine or E2 on the membrane potential of ON cells. In the 5 ON cells tested, E2 consistently depolarized the membrane potential and caused significant increases in the firing rate (Figure 2H), whereas morphine treatment resulted in hyperpolarization and a decrease in the discharge rate (Supplemental Figure 5H).

The observations so far suggested that ON cells exclusively expressed GPER and that GPER might be exploited for the identification and selective manipulation of RVM ON cells. To test this hypothesis, we obtained a *Gper^Cre^* mouse line (28, 30), transfected GPER^+^ neurons with the calcium-sensitive protein GCamp6 [pAAV-EF1a-DIO-GCaMP6(F)-P2A-NLS-tdTomato], and conducted in vivo 2-photon calcium imaging of GPER^+^ neurons through surgical exposure of the ventral medulla (Figure 2I). Successful recordings were made from a total of 63 tdTomato^+^ neurons in 5 mice. A noxious pinch applied to the hind paw was found to evoke transient calcium responses in these neurons (Figure 2, J and K).

### GPER^+^ neurons in the RVM play significant roles in descending regulation of pain.

**Figure 3:**
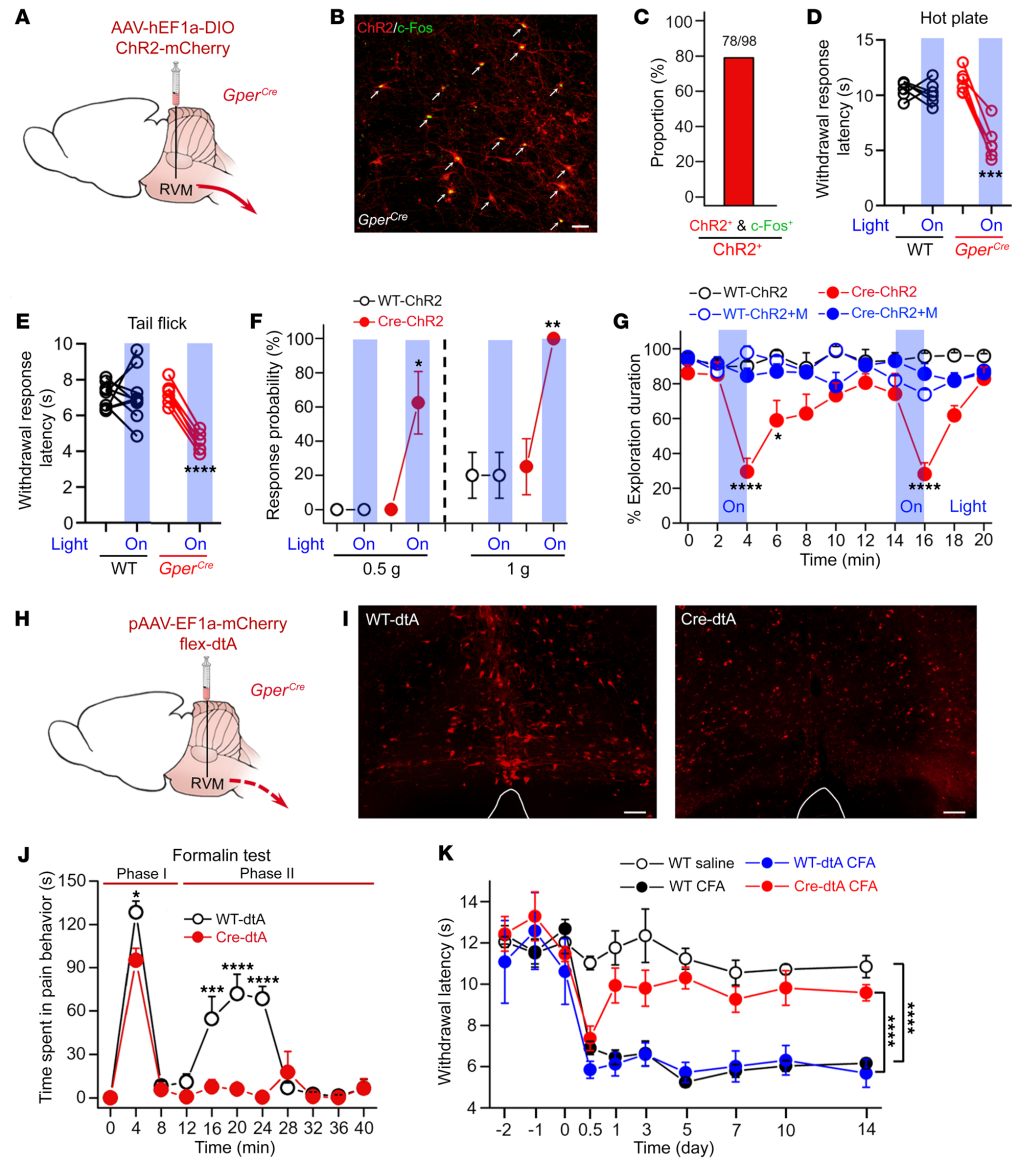
GPER^+^ neurons in the RVM play significant roles in descending regulation of pain. (**A**) Schematic of the experimental approach for optogenetic activation of RVM GPER^+^ neurons using *Gper^Cre^* mice. (**B** and **C**) Light stimulation of the RVM caused c-Fos expression in the majority (78 of 98 neurons) of ChR2^+^ neurons. Scale bar: 50 μm. (**D**–**F**) Light stimulation of GPER^+^ neurons in the RVM significantly increased sensitivity to thermal (hot-plate and tail-flick) and mechanical (**F**) stimulation. *n* = 6–10 mice/group. (**G**) Light stimulation of GPER^+^ neurons caused a significant decrease in spontaneous exploration activity of *Gper^Cre^* mice, which could be reversed by i.p. morphine pretreatment. *n* = 6 mice/group. (**H**) Schematic of the experimental approach for selective ablation of RVM GPER^+^ neurons using the Cre-dependent dtA virus in *Gper^Cre^* mice. (**I**) Cre-dtA mice had a lack of intact mCherry-labeled neurons in the RVM compared with the WT-dtA group. Scale bars: 100 μm. (**J**) The second phase of formalin-induced nociception behaviors was diminished in the Cre-dtA group compared with the WT-dtA group. *n* = 6–11 mice/group. (**K**) CFA-induced chronic pain was diminished in Cre-dtA mice compared with WT-dtA mice. *n* = 7–11 mice/group. Data are representative of at least 3 independent experiments and are presented as the mean ± SEM. *P < 0.05, **P < 0.01, ***P < 0.001, and ****P < 0.0001, by paired, 2-tailed Student’s *t* test (**D**–**F**), 2-way ANOVA followed by Bonferroni’s post hoc test (**G** and **J**), and 1-way ANOVA with Tukey’s post hoc test (**K**).

Next, we investigated the possible effects of selective manipulation of GPER^+^ RVM neurons on pain using the *Gper^Cre^* mouse line. We started with optogenetic activation of RVM GPER^+^ neurons (with Cre-driven expression of ChR2 following microinjection of AAV-hEF1a-DIO-ChR2-mCherry viruses to the RVM, Figure 3A). As shown in Figure 3, B and C, optical stimulation directed to the RVM selectively activated ChR2-expressing neurons (i.e., GPER^+^ neurons), as evidenced by marked c-Fos expression in these neurons. We found that optogenetic activation resulted in significant sensitization of the withdrawal responses to noxious thermal stimulation in the hot plate and in tail-flick tests in the *Gper^Cre^* mice but not in the WT mice injected with the same virus (Figure 3, D and E). Similarly, optogenetic activation of RVM GPER^+^ neurons sensitized the paw withdrawal responses to mechanical probing with von Frey filaments (0.5 g and 1 g) in *Gper^Cre^*, but not WT, mice (Figure 3F). It was also noticed that *Gper^Cre^* mice displayed freezing-like behaviors (marked decreases in exploration activity) during optogenetic stimulation of the RVM. We suspect that such behaviors might be nociception-related, since morphine (5 mg/kg, i.p.) reversed this effect (Figure 3G).

We then studied the effects of selective ablation of GPER^+^ RVM neurons on acute and chronic pain by microinjection of the pAAV-EF1a-mCherry-flex-DTA virus into the RVM of *Gper^Cre^* (Cre-dtA) and WT mice (WT-dtA, Figure 3H). Cells infected with diphtheria toxin A (dTA) virus would nonselectively express mCherry, whereas only GPER^+^ neurons would express DTA, leading to cell death. As expected, 3 weeks after virus injection, numerous “healthy” mCherry-labeled cells could be seen in the RVM of WT-dtA mice, whereas in the Cre-dtA mice, mCherry expression was visible in RVM but cells appeared to be largely disintegrated (Figure 3I). In the formalin test, the first phase of nociception responses (time spent licking and flinching) was not significantly different between Cre-dtA and WT-dtA mice. However, the second phase of nociception responses nearly disappeared in the Cre-dtA group (Figure 3J), suggesting that selective ablation of GPER^+^ RVM neurons may diminish formalin-induced sustained pain. In a chronic inflammatory pain model, compared with the WT-dtA mice, which exhibited sustained hypersensitivity to noxious thermal stimulation following intraplantar injection of complete Freud’s adjuvant (CFA), the latency of heat-induced withdrawal responses of Cre-dtA mice was significantly longer except on day 1 (Figure 3K).

Additionally, we investigated the effects of selective chemogenetic inhibition of GPER^+^ RVM neurons on pain by microinjection of the rAAV-hSyn-DIO-hM4D(Gi)-EGFP virus into the RVM in *Gper^Cre^* and WT mice (Supplemental Figure 6, B and C). Mechanical pain (paw withdrawal response induced by mechanical probing with von Frey hairs) and formalin-induced pain behaviors were tested 30 minutes following administration of the hM4Di agonist clozapine-*N*-oxide (CNO) (0.5 mg/kg, i.p.). We found that administration of CNO resulted in decreased sensitivity of the paw withdrawal responses in the *Gper^Cre^* mice but not in the WT mice injected with the same virus (Supplemental Figure 6, D and E). In the formalin test, both the first phase and the second phase of pain behaviors were significantly inhibited in the Cre-hM4Di group compared with the WT-hM4Di group (Supplemental Figure 6F).

Thus, selective activation of GPER^+^ RVM neurons led to facilitation of acute pain, whereas selective ablation or inhibition of these neurons inhibited acute and chronic pain.

### GPER drives descending facilitation of pain and morphine tolerance.

Our results so far indicated that GPER identifies a subset, the GABAergic ON cells, in the RVM. Besides, activation of GPER in the RVM may significantly increase ON cell excitability and decrease the analgesic effect of local morphine. We next sought to determine the significance of GPER signaling in pathological pain, morphine analgesia, and tolerance, given that the RVM has been implicated in chronic pain conditions and also recognized as an important site for opioid analgesia (31).

**Figure 4:**
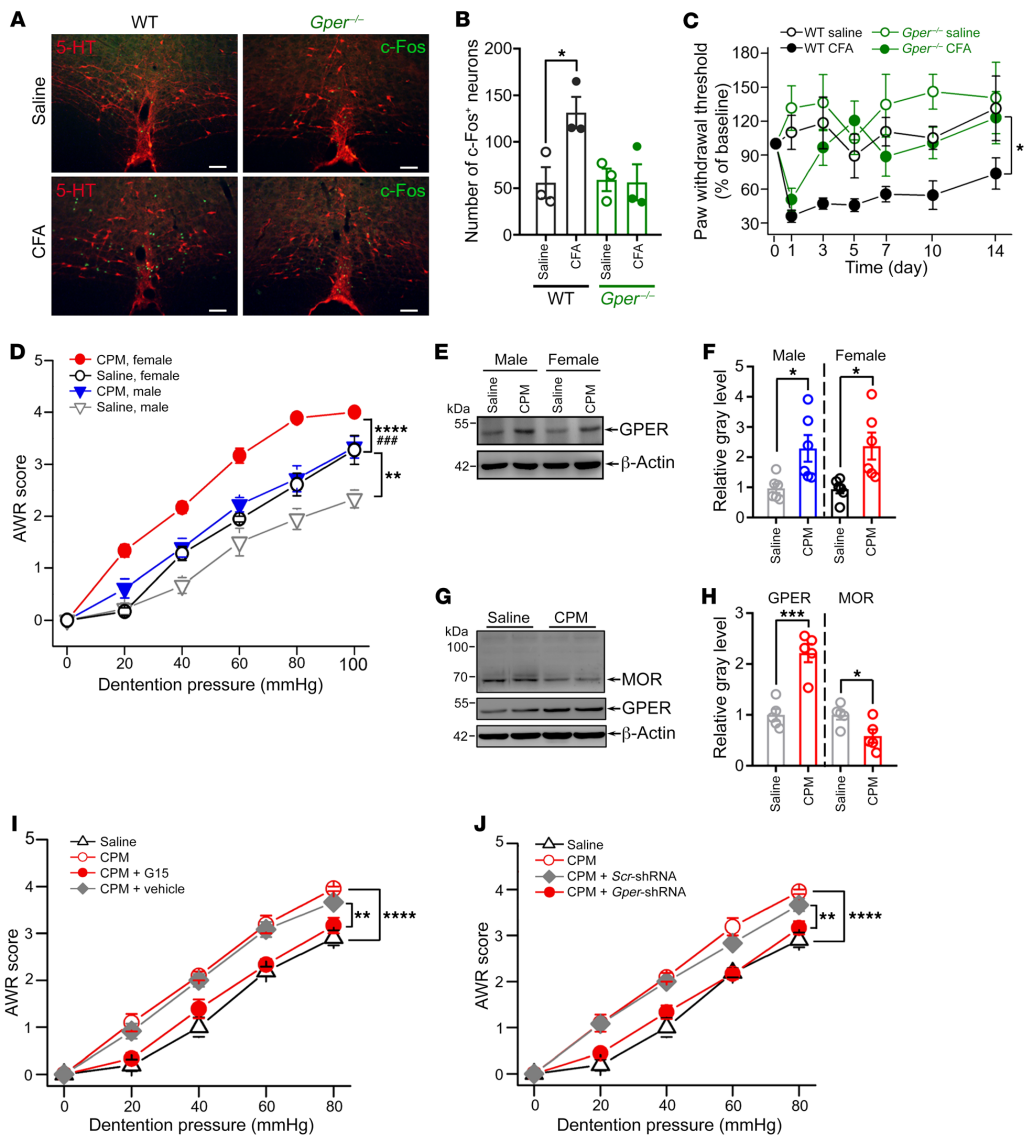
GPER drives descending facilitation of somatic and visceral pain. (**A** and **B**) c-Fos immunofluorescence showing activation of RVM nonserotonergic neurons following CFA treatment in WT but not *Gper^–/–^* female rats. *n* = 3 rats/group. Scale bars: 100 μm. (**C**) CFA treatment caused sustained hypersensitivity of the paw withdrawal responses to von Frey stimulation in WT female rats, while *Gper^–/–^* female rats displayed only a transient hypersensitivity on day 1 following CFA injection. *n* = 5–8 rats. (**D**) Repeated administration of CPM caused colorectal hypersensitivity in female and male rats. Note that the female rats showed higher sensitivity to CRD than did male rats. *n* = 6 rats/group. (**E** and **F**) GPER expression in the RVM was increased in CPM-treated female and male rats compared with saline-treated controls. *n* = 6 rats/group. (**G** and **H**) MOR expression in the RVM was decreased, with a concomitant increase in GPER expression in CPM-treated female rats compared with saline-treated female rats. *n* = 5 rats/group. (**I** and **J**) Chronic intracisternal infusion of G15 or *Gper* gene knockdown by local injection of shRNA-AAV both significantly alleviated CPM-induced colorectal hypersensitivity. *n* = 4–7 rats/group. Data are representative of at least 2 independent experiments. Data are presented as the mean ± SEM. *P < 0.05, **P < 0.01, ***P < 0.001, ^###^*P* < 0.001, and ****P < 0.0001, by unpaired, 2-tailed Student’s *t* test (**B**, **F**, and **H**) and 1-way ANOVA with Tukey’s post hoc test (**C**, **D**, **I**, and **J**).

We obtained a line of *Gper*-knockout rats (28, 30), which showed a significant reduction of GPER immunofluorescence in the RVM (Supplemental Figure 7). We compared the level of RVM neuronal activity between WT and *Gper^–/–^* rats in chronic inflammatory pain conditions by c-Fos immunostaining conducted 7 days after intraplantar injection of CFA (with saline as a control). We detected a significant increase in the number of c-Fos^+^ neurons in the RVM, concomitant with a sustained mechanical hypersensitivity in CFA-treated rats compared with saline-treated WT rats (Figure 4, A–C). Importantly, c-Fos^+^ neurons were almost exclusively nonserotonergic neurons (Figure 4A), indicating that activation of nonserotonergic RVM neurons contributed to chronic inflammatory pain. Strikingly, c-Fos activation was not observed in CFA-treated *Gper^–/–^* rats, which concomitantly only displayed transient mechanical hypersensitivity on day 1 (Figure 4, A–C).

Repeated application of cyclophosphamide (CPM) is known to result in colorectal hypersensitivity due to central sensitization (32). We replicated this model in male and female rats and found that colorectal hypersensitivity was associated with increased GPER and decreased μ-type opioid receptor (MOR) expression in the RVM (Figure 4, D–H). Microinjection of a low dose of G15 (10 μM, 0.5 μL) into the RVM significantly attenuated the CRD-induced VMR in CPM-treated rats but not in naive rats (Supplemental Figure 8, A and B). These results suggested that altered GPER and opioid signaling might contribute to visceral hyperalgesia in this model. We further addressed this possibility by chronic intracisternal administration of G15 (through osmotic minipumps) or by knocking down GPER expression in the RVM (through microinjection of *Gper* shRNA adeno-associated virus [AAV], Supplemental Figure 8, C and D) before CPM application. Consistently, we found that chronic intracisternal application of G15 or *Gper* knockdown both prevented the development of colorectal hyperalgesia following repeated application of CPM (Figure 4, I and J).

The results obtained from the CPM and CFA models strongly suggest that GPER signaling in the RVM may play a crucial role in the maintenance of chronic pain.

**Figure 5:**
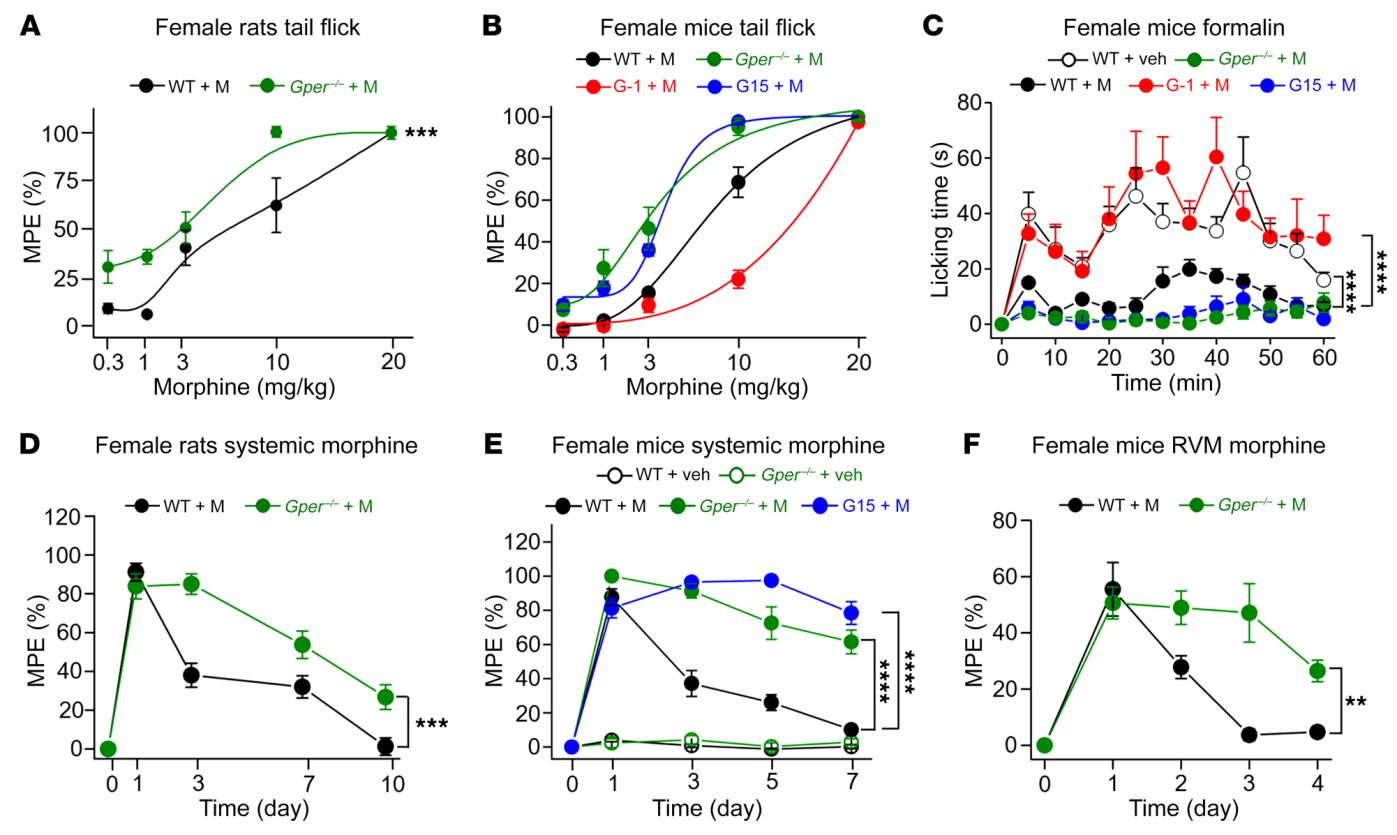
GPER inhibits morphine analgesia and promotes morphine tolerance. (**A**) Analgesic efficacy, expressed as a percentage of the maximal possible efficiency (MPE) of systemic morphine (M), was increased in *Gper^–/–^* female rats compared with WT female rats in the tail-flick test. *n* = 7 rats/group. (**B** and **C**) The analgesic efficacy of systemic morphine was decreased following activation of GPER with G-1 (1 mg/kg, i.p., 30 minutes prior to the test), whereas blockade of GPER with G15 or *Gper* deficiency led to enhanced analgesia in the tail-flick and formalin tests in female mice. *n* = 6–9 mice. (**D**) The development of tolerance to systemic morphine was slowed in *Gper^–/–^* female rats compared with WT female rats. *n* = 6 rats/group. (**E**) The development of tolerance to systemic morphine was impaired in *Gper^–/–^* female mice compared with WT female mice. Blockade of GPER with G15 also prevented the development of tolerance to systemic morphine in WT female mice. *n* = 6 mice/group. (**F**) The development of tolerance to morphine microinjected into the RVM was significantly slowed in *Gper^–/–^* female mice compared with WT female mice. *n* = 6 mice/group. Data are representative of at least 3 independent experiments and are presented as the mean ± SEM. **P < 0.01, ***P < 0.001, and ****P < 0.0001, by unpaired, 2-tailed Student’s *t* test (**A**, **D**, and **F**) and 1-way ANOVA with Tukey’s post hoc test (**C** and **E**).

The endogenous opioid system plays a vital role in pain regulation at least partly through actions on the descending pain modulation systems (31). Our initial observation that E2 applied to the RVM not only augmented visceral pain but also diminished morphine analgesia implies that GPER may profoundly affect opioid signaling. To test this hypothesis further, we examined whether morphine analgesia would be affected in *Gper* deficiency or following pharmacological activation or antagonism of GPER. In the tail-flick test, the systemic morphine dose-response curve for female *Gper^–/–^* rats was shifted to the left compared with that for female WT rats (Figure 5A). In the WT female mice, activation of GPER by G-1 significantly decreased the analgesic efficacy of systemic morphine, causing a rightward shift of the dose-response curve. In contrast, the GPER antagonist G15 or *Gper* deficiency markedly enhanced morphine analgesia, resulting in a leftward shift of the morphine dose-response curve (Figure 5B). Similarly, in the formalin test, morphine (3 mg/kg) analgesia was significantly enhanced in the presence of G15 and in *Gper^–/–^* mice (Figure 5C). Therefore, activation of GPER inhibited opioid analgesia, whereas antagonism or genetic ablation of GPER may enhance it.

These results raised the possibility that GPER might also regulate the development of opioid tolerance, which manifests as decreased analgesic efficacy following repeated use of these drugs. To test this hypothesis, female WT and *Gper^–/–^* rats were treated with morphine (10 mg/kg, s.c. injection) daily to observe its efficacy in tail-flick response latency. In the female WT rats, morphine produced potent analgesia on day 1, but the analgesic effect decreased significantly after day 3, indicating the development of tolerance, while the development of tolerance was apparently delayed in the *Gper^–/–^* rats (Figure 5D). More strikingly, systemic morphine analgesia was largely maintained throughout the test period (7 days) in female *Gper^–/–^* mice, in contrast to female WT mice, in which the analgesic efficacy of morphine decreased significantly starting on day 3 (Figure 5E). Coadministration of the GPER antagonist G15 also prevented the development of morphine tolerance in WT mice. Morphine (2 μg in 0.5 μL) microinjected into the RVM produced potent analgesia on day 1, but this effect declined significantly between days 2 and 4. In *Gper^–/–^* mice, the analgesic effect of morphine administered to the RVM remained similar over the 4-day test period (Figure 5F). Therefore, both pharmacological antagonism and genetic ablation of GPER may significantly delay the development of morphine tolerance.

We also conducted similar types of experiments in male animals to observe the effects of *Gper* deficiency on morphine analgesia. We found that CFA-induced chronic inflammatory pain was attenuated in male *Gper^–/–^* rats, with enhanced morphine analgesia, compared with male WT rats (Supplemental Figure 9A). Morphine analgesia was also enhanced in male *Gper^–/–^* mice compared with male WT mice (Supplemental Figure 9, B and C). The development of morphine tolerance was also delayed in male *Gper^–/–^* mice compared with male WT mice (Supplemental Figure 9, D and E).

### Activation of GPER inhibits opioid signaling in ON cells.

**Figure 6:**
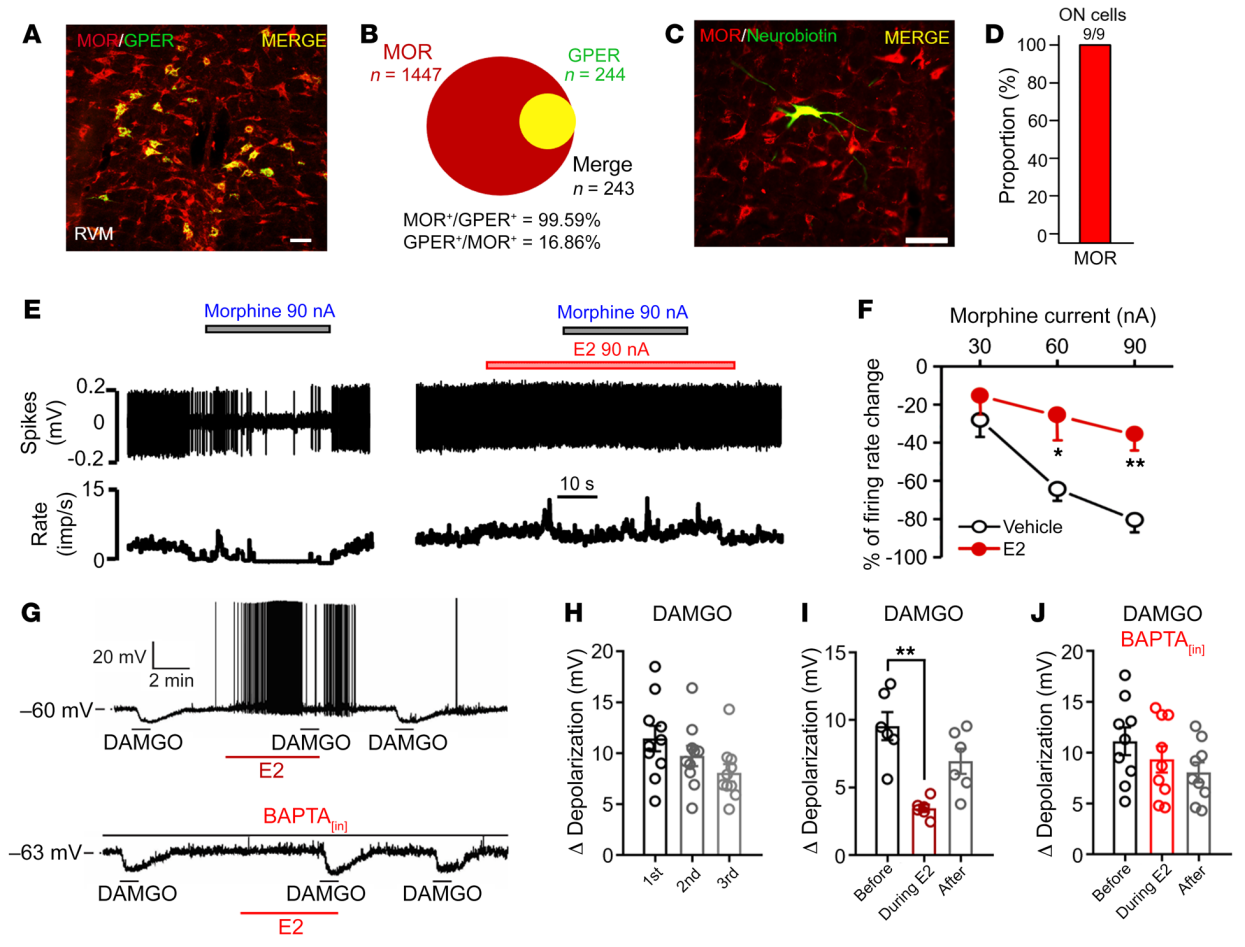
Activation of GPER inhibits μ-opioid signaling in a calcium-dependent manner. (**A**) Immunofluorescence showing coexpression of MOR and GPER in the RVM of female rats. Scale bar: 50 μm. (**B**) Percentage of neurons coexpressing MOR and GPER in the RVM of WT rats. (**C** and **D**) Electrophysiologically identified RVM ON cells (*n* = 9) were all immunoreactive to MOR. Scale bar: 100 μm. (**E** and **F**) Morphine-induced inhibition of RVM ON cells was diminished by prior treatment with E2 in the rat in vivo. *n* = 5–6 cells/group. (**G** to **J**) GPER activation with E2 negated DAMGO-induced hyperpolarization in medulla slices in vitro. The effect of E2 was calcium dependent, since the presence of BAPTA (a calcium chelator) in the pipette solution prevented this effect. *n* = 6–10 cells/group. Data are representative of at least 3 independent experiments and are presented as the mean ± SEM. *P < 0.05 and **P < 0.01, by 2-way ANOVA followed by Bonferroni’s post hoc test (**F**) and paired, 2-tailed Student’s *t* test (**I**).

Morphine mediates analgesia in the RVM by activating MOR, which is coupled with activation of the G protein–regulated inward-rectifying potassium channel (GIRK) with consequent membrane hyperpolarization and inhibition of ON cells (33, 34). Previous studies have shown that estrogens may uncouple G_i/o_-GPCRs (e.g., MOR and GABA_B_ receptors) from activating the GIRK (35), while we have found that GPER agonists (E2 or G-1) depolarized RVM neurons in vitro (Figure 1) and in vivo (Figure 2). These data raised the possibility that GPER may desensitize MOR-mediated activation of potassium channels, with consequent activation of RVM ON cells. Consistently, we found that 99.59% of GPER^+^ neurons were also strongly immunoreactive for MOR, although only 16.86% of MOR^+^ neurons showed strong GPER immunoreactivity (Figure 6, A and B). Furthermore, 9 of 9 ON cells functionally identified in vivo were also immunoreactive for MOR (Figure 6, C and D). Extracellular recordings of RVM neurons with multibarrel electrodes showed that morphine applied by iontophoresis suppressed the action potential firing of functionally identified ON cells, and the effect was greatly attenuated in the presence of E2 (Figure 6, E and F). In medulla slices, (d-Ala2, *N*-Me-Phe4, Gly5-ol)-enkephalin (DAMGO), a MOR agonist, was found to induce hyperpolarization of the membrane potential (e.g., Figure 6G), and this effect was largely reproducible over repeated DAMGO exposures (Figure 6H). However, DAMGO-induced hyperpolarization of RVM neurons was negated by the GPER agonist E2 (Figure 6I). These results clearly indicate that activation of GPER may suppress μ-opioid signaling.

How does GPER activation lead to inhibition of MOR signaling? Previous studies in hypothalamic proopiomelanocortin (POMC) neurons indicate that E2 may inhibit Gi/o-GPCR signaling through the calcium and PKC/PKA cascades, but the E2 receptor mediating such an effect was not clear (35, 36). We noticed that GPER seemed to be distinctively located on the Golgi apparatus (Supplemental Figure 10), which is a calcium store besides being the site of protein processing (37). These data prompted us to investigate the possibility that calcium might be involved in GPER-mediated inhibition of MOR signaling. In the medulla slice, the Ca^2+^ chelator 1,2-bis(*o*-aminophenoxy)ethane-*N*,*N*,*N*′,*N*′-tetra-acetic acid (BAPTA) (in pipette solution) negated GPER-mediated inhibition of DAMGO currents (Figure 6, G and J). Furthermore, we recently demonstrated that in neuroblastoma SH-SY5Y cells, which endogenously express GPER and MOR, activation of GPER led to rapid calcium release with subsequent PKC activation and MOR phosphorylation (38). Collectively, these data indicate that activation of GPER may suppress MOR signaling in a calcium/PKC-dependent manner.

## Discussion

### GPER identifies a population of RVM ON cells.

Chronic pain affects a vast number of people worldwide. In 2016, an estimated 20.4% (50 million) of adults had chronic pain, and 8.0% of adults (19.6 million) had high-impact chronic pain in the United States, with a greater prevalence of both chronic pain and high-impact chronic pain reported among women (39). In the United Kingdom, chronic pain affects between one-third and one-half of the population, also with a higher prevalence in women (40). However, current treatments for chronic pain are limited, ineffective, and addictive (41). A better understanding of the mechanisms underlying pain and analgesic tolerance is crucial for the development of new therapies.

Recent neuroimaging studies conducted in humans indicate that functional connectivity of the RVM is altered in various forms of chronic pain and in opioid analgesic users (13–16). However, our understanding of cellular organization and signaling mechanisms within the RVM in normal and chronic pain conditions is still limited. In the current study, we detected distinct expression of GPER in rat RVM. Several complementary approaches were taken to determine which cell types express GPER. We found that GPER^+^ neurons were nonserotonergic but expressed GAD67 and MOR. They were spinally projecting neurons and inhibited by MOR agonists. Importantly, functionally identified, intracellularly labeled ON cells, but not OFF or neutral cells, were immunoreactive to GPER. GPER agonists selectively activated ON, but not OFF, neurons. Furthermore, using *Gper^Cre^* mice, we found that GPER^+^ neurons exhibited typical ON-like responses following noxious peripheral stimulation. Optogenetic activation of GPER^+^ neurons facilitated pain, while ablation or inhibition of GPER^+^ neurons ameliorated pain. These findings have provided direct evidence that GPER identifies a population of GABAergic ON cells.

Earlier in vivo electrophysiological studies have shown that in the RVM, ON cells exclusively respond to MOR agonists (42), so MOR expression might be considered a hallmark of RVM ON cells, although there has been evidence that OFF or neutral cells may also express MOR (12, 42, 43). Our IHC experiment showed that only a minority (~16.8%) of MOR^+^ neurons in the RVM were GPER^+^. We think that 16.8% might be an underestimate of the proportion of ON cells that express GPER, given the methodological limitations, such that ON cells with weaker GPER immunoreactivity might be missed. Nevertheless, GPER^+^ neurons likely represent a subset, that of GABAergic ON cells. These long-range GPER^+^ GABAergic ON cells likely facilitate pain transmission in the spinal cord by inhibiting enkephalinergic and GABAergic interneurons (19). It is likely that they play a general, modality-independent role in pain regulation, since we found that GPER^+^ neurons were activated by different noxious stimulations, visceral or somatic, and their activation or deactivation affected behavior responses in diverse pain models. However, GPER^+^ neurons may not be required to maintain baseline nociceptive responses in the normal physiological state, rather, they can be engaged by noxious visceral or somatic afferent signals and in turn participate in the genesis and maintenance of pathological pain.

Serotonergic neurons constitute approximately one-fourth of the spinally projecting neuronal population within the RVM. However, their functions in pain modulation remain ambiguous, as both anti- and pronociceptive roles have been reported for this group of neurons (44). Intrathecal administration of 5-HT can produce antinociception (45). However, optogenetic activation of serotonergic neurons induces prolonged hyperalgesia (46). The nuanced role of RVM serotonergic neurons may partly be explained by different classes of 5-HT receptors existing in the spinal dorsal horn (47). However, it is also possible that serotonergic neurons may affect the excitability of ON and OFF neurons in addition to projecting directly to the spinal cord (48). In this study, we found that GPER was exclusively detected in nonserotonergic neurons and that numerous varicose 5-HT^+^ fibers were in close proximity with GPER^+^ soma, suggesting that serotonergic neuron activity may influence descending facilitation.

It was interesting to note that the distribution of GPER^+^ neurons was somewhat different between male and female rats. In the female rats, GPER^+^ neurons were very much confined to the raphe nuclei, but in male rats more GPER^+^ neurons were also found in the gigantocellular reticular nucleus, GiA and GiV parts. This finding suggests that the cellular organization of the RVM is sex dimorphic, which may contribute to the sex differences in pain and analgesia.

### GPER mediates hormonal regulation of descending facilitation and opioid signaling.

We undertook this study because of the significant sex differences in the experience of pain and analgesia and the overwhelming evidence indicating that females are more susceptible to pain and less sensitive to opioid analgesia (49, 50). Female hormones may therefore play important roles in the regulation of pain transmission, modulation, and perception as well as of opioid signaling.

Previous reports indicate that the anatomical and functional organization of the PAG-RVM system is sexually dimorphic and that morphine preferentially activates the PAG-RVM pathway in the male rat (51). In the current study, we directly investigated the influence of estrogens applied to the RVM on pain. Remarkably, we found that E2 applied to the RVM (or intracisternally) rapidly potentiated nociception and blocked morphine analgesia. We determined that such effects were mediated by GPER rather than by the nuclear receptors ERα or ERβ. This conclusion was based on multiple lines of evidence: (a) GPER immunoreactivity was distinctively abundant compared with ERα or ERβ immunoreactivity within the RVM; (b) the effects of E2 occurred within seconds, which could not be explained by the nuclear receptor–mediated genomic actions; (c) the effects were mimicked by the GPER-selective agonist G-1 and blocked by the GPER antagonist G15 but not ERα or ERβ antagonists; (d) E2 produced rapid membrane depolarization of RVM neurons in medulla slices from WT but not *Gper^–/–^* rats. Together with previous reports, our findings indicate that the female hormone estrogens may profoundly influence the activity of the PAG-RVM system.

Opioid signaling plays dominant roles in the control of descending modulation of pain (8, 19). The MOR is widely distributed along the descending system including the PAG and the RVM (12, 52, 53), therefore, the level of MOR activation may determine the output of the PAG/RVM pathway. Within the RVM, opioids such as morphine produce analgesic effects by hyperpolarizing ON cells through activation of GIRK channels (33, 34). We argue that GPER is a major player in the regulation of MOR signaling within the RVM. This is evident from the following results obtained in vivo and in vitro: (a) morphine microinjected into the RVM produced powerful analgesic effects by silencing ON cell firing, an effect that was virtually abolished by prior local administration of E2; (b) electrophysiologically identified ON cells coexpressed GPER and MOR and were rapidly depolarized by E2; (c) in the medullary slice in vitro, MOR agonist DAMGO caused membrane hyperpolarization of RVM neurons, which was negated in the presence of E2; (d) also in the medullary slice, E2 or G-1 caused GPER-dependent rapid membrane depolarization of RVM neurons. We have previously shown that aromatase, the enzyme responsible for the transformation of testosterone into estradiol, is abundant and upregulated within the RVM in chronic colorectal hyperalgesia (32). Here, GPER expression was found to be increased in chronic colorectal hyperalgesia and CFA-induced chronic inflammatory pain in the rat. Moreover, either chronic intracisternal infusion of G15 or siRNA knockdown of GPER within the RVM was sufficient to reverse CPM-induced chronic colorectal hyperalgesia. CFA-induced chronic inflammatory pain was also diminished in *Gper*-deficient rats and mice. These data together suggest that circulating or locally produced estrogens may increase the tone of descending facilitation through GPER-mediated disinhibition of ON cells from opioid inputs, thereby contributing to the development or maintenance of visceral or somatic pain.

Opioids remain the most effective treatment for pain, but the rapid development of tolerance and addiction to opioids poses a significant challenge (54). The current study provides evidence that GPER-mediated inhibition of MOR signaling is an important mechanism underlying opioid tolerance. Accordingly, the analgesic efficacy of systematic morphine was markedly enhanced, and development of tolerance was impaired in *Gper^–/–^* rats and mice compared with that observed in the WT animals. Importantly, although systematic morphine produces analgesia at multiple levels of the neuraxis, the RVM is indispensable for full analgesic efficacy of morphine (55). We found that the development of tolerance to morphine analgesia within the RVM was also impaired in *Gper^–/–^* rats and mice. These results together raise the possibility that GPER antagonism may be a potential means to enhance opioid analgesia and prevent the development of tolerance.

This study has some important limitations that warrant consideration. The concentrations of 17β-E2 applied to RVM neurons in vitro were between 0.1 and 1 μM, which is much higher than plasma E2 concentrations. This raises the question of how relevant E2-mediated depolarization is in physiology or pathology. It should be noted that E2 was applied by a pipette as a point source placed some distance away from the cell being recorded, so that E2 might be diluted by the bath solution. Additionally, E2 had to diffuse some distance through the overlying tissue to act upon the cell. For these reasons, the actual concentration of E2 acting on the cell could be much lower than the pipette concentrations. The local concentration of E2 in the RVM is not known, however, it is worth noting that the brain may accumulate and synthesize E2 de novo (56). As we have previously shown that the RVM is enriched with aromatase (32), it may be interesting to test whether GPER^+^ neurons express aromatase.

Another important limitation of this study is that germline, systematic *Gper*-knockout rats and mice were used to study the role of GPER signaling in the regulation of pain and analgesia. This raises the question of how specific the effects were to GPER signaling in the RVM. We have previously reported that *Gper^–/–^* rats showed increased anxiety (28). Therefore, we cannot rule out the possibility that the differences between *Gper^–/–^* and WT animals could be the result of altered “top-down” recruitment of ON-cells rather than from a direct action of GPER in the RVM. Nevertheless, it is conceivable that GPER in the RVM is at least partially responsible for the observed phenotypes.

In summary, the current study revealed distinctive GPER expression in a population of GABAergic ON cells in the RVM. On the basis of the current and previously reported data, we propose that, at the circuit level, activation of GPER mediates disinhibition of ON cells from opioidergic inputs and that increased activity of GABAergic ON cells may decrease the activity of inhibitory interneurons in the spinal cord (19), resulting in disinhibition of nociceptive neurons and facilitated ascending pain transmission. At the molecular level, exogenous and endogenous opioids interact with MOR, which is coupled to GIRK channels, causing hyperpolarization of ON cells and analgesia. Estrogens interact with GPER to promote MOR phosphorylation via the Ca^2+^/PKC pathway (30, 38) and decouple MOR from the GIRK, which may lead to increased activity of ON cells, enhanced pain, and opioid tolerance.

## Methods

### Animals

WT and transgenic Sprague-Dawley rats (male and female, either 10- to 12-week-old adult rats weighing 220–250 g or P12- to 15-day-old neonatal rats) and C57/BL6 mice were housed (4 animals per cage) in a temperature-controlled (22°C–25°C) and humidity-controlled (50%) room with a 12-hour light/12-hour dark cycle and had ad libitum access to food and water. *Gper*-deficient (*Gper^–/–^*) rats (28) and mice (30) were generated by the Bioray Biotechnology Company in Shanghai. *Gper^Cre^*-transgenic mice (30) were generated by the Shanghai Biomodel Organism Science and Technology Development Company by knocking the 2A-Cre gene fragment into the *Gper* gene stop codon using the CRISPR/Cas9 system.

### In vivo electrophysiology in rats

Adult female rats were anesthetized with an i.p. injection of sodium pentobarbital (60 mg/kg). The trachea was intubated to assist spontaneous ventilation. A pair of electromyography (EMG) electrodes made with Teflon-coated stainless-steel wires were implanted into the left internal oblique muscle of the abdomen to record the VMR to noxious CRD. For CRD, a polyethylene balloon with a connecting catheter was prepared and inserted into the distal colon (2 cm from the anus). The catheter was fixed to the tail with tape. With a T-shaped connector, the catheter was connected to a water reservoir/infusion pump and pressure transducers. The animal was then mounted onto the stereotaxic apparatus in a supine position, and light anesthesia was maintained by inhalation of 2% isoflurane. A craniectomy was performed to allow access of microinjection needles or recording electrodes to the RVM according to the rat brain atlas.

### Recording of the CRD-induced VMR

EMG of abdominal muscles was recorded using a Neurolog system (Digitimer Ltd.). Either phasic CRD with a water reservoir (60 mmHg for 30 seconds) or ramp CRD with an infusion pump (pressure rising from 0 to 60 mmHg within 30 seconds) was repeated at 3- or 5-minute intervals. The EMG signal and the distension pressure signal were digitized using Power 1401 (Cambridge Electronic Design) and analyzed using Spike2 software (Cambridge Electronic Design [CED]). Drugs (E2, G-1, morphine or G15) were diluted in artificial cerebral spinal fluid (aCSF) to the required concentration (E2: 0.01 μM, 0.1 μM, and 1 μM, respectively; G-1: 1 μM; morphine: 10 mM and G15: 10 μM and 50 μM) and microinjected into the RVM (–10.3 mm posterior and –8.7 mm deep from bregma, 0 mm lateral from the midline) in a volume of 0.5 μL. The vehicle control experiment was conducted by injecting equal volume of aCSF.

### Extracellular recording and functional identification of RVM neurons

For the in vivo extracellular recording and drugs application, 6-barrel electrodes were prepared in-house. One of the barrels was filled with 3 M NaCl for recording (electrode resistance of 3–5 MΩ). Another barrel was filled with 2 M NaCl to serve as the current balance barrel. The other barrels were filled with 100 mM glutamate, 20 mM 17β-estradiol sodium sulfate, or 26.6 mM morphine hydrochloride (electrode resistance between 60 and 80 MΩ). A recording was made in the RVM area using the following coordinates according to the rat brain atlas: 10.0–13.0 mm caudal to bregma; –1.0–1.0 mm lateral to midline; and 8.8–10.0 mm from cerebellum dura. The electrical signal was amplified and filtered using the Neurolog system (Digitimer), digitized using Power 1401, and analyzed using Spike2 software. After a stable extracellular recording was established, the neurons were tested for their responses to noxious CRD or noxious pinch applied to the hind paw. Cells that exhibited excitatory responses were considered ON neurons, while those showing inhibitory responses were considered OFF neurons. Drugs were applied by passing appropriate currents to the respective barrels using the Micro-Iontophoresis Current Generator (6400 ADVANCED, Dagan Corporation). The current balance was affected in an automatic manner.

### Intracellular recording and functional identification and labeling of RVM neurons

Intracellular recordings of RVM neurons were conducted using single sharp electrodes filled with 3 M KCl and 1% Neurobiotin (Vector Laboratories, SP-1120) with a tip resistance of 90–110 MΩ. Neurons with a potential more negative than –40 mV and an action potential amplitude greater than 80 mV were included in the data analysis. ON or OFF neurons were identified on the basis of changes in membrane potential and firing rate in response to noxious CRD and pinch applied to the hind paw, with subsequent iontophoretic injection of Neurobiotin. After completion of the experiment, the animal was euthanized with an overdose of sodium pentobarbital, followed immediately by transcardiac perfusion with saline and 4% paraformaldehyde (PFA). The brain was removed and postfixed overnight in 4% PFA at 4°C for immunohistochemical analysis. In another series of experiments, an intracellular recording was also made using piggyback electrodes (a sharp electrode glued to multibarrel iontophoresis electrodes) to observe the effects of E2 and morphine on the membrane potential and firing rate of functionally identified RVM neurons.

### Brain slice electrophysiology

#### Slice preparation.

RVM slice preparation was performed as described previously with minor modifications (57). Briefly, 12- to 15-day-old rats were deeply anesthetized with isoflurane, and the brain was rapidly removed and placed in oxygenated (95% O_2_/5% CO_2_, v/v) ice-cold aCSF containing 125 mM NaCl, 2.5 mM KCl, 2 mM CaCl_2_, 1 mM MgCl_2_, 1.25 mM NaH_2_PO_4_, 25 mM NaHCO_3_, and 12.5 mM d-glucose (pH 7.35–7.45). Brainstem slices (300 μm) containing RVM tissue were cut with a vibrating microtome (VT 1200S, Leica Microsystems) in oxygenated aCSF at 4°C. The slices were incubated in the aCSF at 31°C for at least 1 hour. A single slice was then placed onto the recording chamber and perfused with oxygenated aCSF at a rate of 2 mL/min.

#### Whole-cell patch-clamp recordings.

Whole-cell patch-clamp recordings in current-clamp mode were made in RVM neurons. The recording pipettes (7–9 MΩ resistance) were pulled by a horizontal micropipette puller (P-97, Sutter Instruments) from borosilicate capillaries and filled with the following internal solution: 120 mM K-gluconate, 10 mM KCl, 5 mM NaCl, 1 mM CaCl_2_ × 2H_2_O, 2 mM MgCl_2_ × 6H_2_O, 11 mM EGTA, 10 mM HEPES, 2 mM Mg-ATP, and 1 mM Li-GTP, with the pH adjusted to 7.3 with Tris base. A 5-minute equilibration period was allowed to reach a steady state after whole-cell access was established. The membrane potential signal was amplified using a MultiClamp 700B (Molecular Devices), filtered at 2 kHz, and digitized at 5 kHz. Data were acquired using pClamp 10.4 software (Molecular Devices). The control solution was continuously delivered by pressure to the soma of each recorded neuron via a 150 μm diameter tip perfusion pipette controlled by a DAD-12 Superfusion System (ALA Scientific Instruments). E2 (100 nM, 300 nM, and 1 μM), G-1 (1 μM), and DAMGO (5 μM) were applied for 3 minutes, 1 minute, and 1 minute, respectively.

### AAV injection

AAV virus carrying *Gper* shRNA was obtained from Neuron Biotech. The *Gper* shRNA sequence was as follows: sense sequence, 5′-CGTCCCAGACCTGTACTTCATTTCAAGAGAATGAAGTACAGGTCTGGGATTTTTTGGAAG-3′; antisense sequence, 5′-AATTCTTCCAAAAAATCCCAGACCTGTACTTCATTCTCTTGAAATGAAGTACAGGTCTGGGACGGGCC-3′. For virus injection, rats were anesthetized with sodium pentobarbital (50 mg/kg, i.p.) and placed in a stereotaxic apparatus. A small hole was drilled into the skull to allow access of the injection needle to the RVM (anteroposterior, –10.3 mm; mediolateral, 0.0 mm; dorsoventral, –8.7 mm with respect to Bregma) according to the Paxinos and Watson Atlas (58). Viral solution (1 μL per rat, 3.64 × 10^12^ vg/mL) was injected into the RVM at a rate of 0.2 μL/min with a Hamilton microsyringe controlled by a programmable infusion pump mounted on the stereotaxic apparatus. The Hamilton microsyringe was removed 5 minutes after microinjection was completed, and the skin incision was closed with a suture. Rats were used for experiments 4 weeks after the injection.

### Two-photon calcium imaging

WT or *Gper^Cre^* mice were preinjected with pAAV-EF1a-DIO-CGaMP6(F)-P2A-NLS-tdTomato (0.5 μL per mouse, 3 × 10^13^ vg/mL) into the RVM, under anesthesia (60 mg/kg sodium pentobarbital, i.p.). Three weeks later, the mice were used for 2-photon imaging of the RVM neurons in the supine position. Mice were anesthetized with 1%–1.5% isoflurane, and then a midline incision was made to expose the trachea, which was then intubated with a U-shaped cannula connected to a small animal ventilator (SomnoSuite, Kent Scientific Corporation). When the mice had stable artificial respiration with the ventilator, the trachea was cut from the oral side. The distal side of trachea with the U-shaped cannula was pulled down to expose the ventral skull. The tissues covering the skull were pulled aside to expose the cranial bone. A small hole was then drilled into the cranial bone to expose the RVM region of the brainstem for 2-photon calcium imaging. The depth of sedation could be adjusted on the small animal ventilator. Calcium fluorescence was observed with a 2-photon microscope (Olympus FVMPE-RS, Olympus). The fluorescence signal was acquired and analyzed using Olympus cellSens Dimension software (version 2.3.1.163). The image size was 512 × 512 pixels or 509.117 × 509.117 μm (unit-converted).

### Optogenetic activation and chemogenetic ablation or inhibition of GPER^+^ neurons

For optogenetic activation of RVM GPER^+^ neurons, we pretreated WT and *Gper^Cre^* mice with a local injection of AAV-hEF1a-DIO-ChR2-mCherry viruses and implanted the optic fiber into the RVM. Three weeks later, mice were habituated to the stimulation apparatus for 1 hour a day for 3 days. Blue light (473 nm) was delivered in 10 ms pulses at 20 Hz, with a 2-minute lights-on period following a 10-minute lights-off period, and this was repeated 2 times over a total period of 30 minutes. The spontaneous behavior and evoked (mechanical or thermal) pain responses were observed before, during, and after light stimulation. Chemogenetic ablation or inhibition of GPER^+^ neurons was done by microinjection of pAAV-EF1a-mCherry-flex-DTA or rAAV-hSyn-DIO-hM4D(Gi)-EGFP viruses into the RVM of *Gper^Cre^* mice (WT mice were used as a control). Ablation efficacy was confirmed by the absence of intact mCherry^+^ neuronal bodies in the RVM of *Gper^Cre^* mice.

### Pain behaviors and chronic pain models

#### CPM-induced cystitis.

Rats were i.p. injected with CPM (MilliporeSigma, C0768, 50 mg/kg) every 3 days to induce chronic cystitis. Control rats received saline injections. At day 17, colorectal sensitivity was measured by recording the response to CRD as described in detail previously (59). Briefly, all animals were habituated to the test environment for 3 days before measurement. On the day of the behavior test, rats were lightly anesthetized with isoflurane. A plastic balloon attached to tygon tubing was inserted 6 cm into the colorectum via the anus and fixed by taping the tubing to the tail. Rats were placed in small lucite cubicles (20 × 8 × 8 cm) and allowed to adapt for 30 minutes. CRD was performed by rapidly inflating the balloon using a sphygmomanometer. The balloon was inflated to various pressures (10, 20, 40, 60, 80, and 100 mmHg) for 20 seconds, followed by a 4-minute rest. Abdominal withdrawal reflex (AWR) responses to each CRD were observed by an operator blinded to the distension pressure and treatment (saline, CPM, CPM plus G15, or CPM plus vehicle). The AWR was scored as follows: 0 = normal behavior; 1 = slight head movement without an abdominal response; 2 = contraction of the abdominal muscles; 3 = lifting of the abdominal wall; or 4 = body arching and lifting of the pelvis. Each measurement was performed 3 times, and the repetitive AWR scores for each distension pressure were averaged.

In order to study the effects of GPER blockade on CPM-induced colorectal hypersensitivity, the GPER antagonist G15 (1 mM at the rate of 0.5 μL/h) was chronically infused intracisternally. In brief, after the dura mater between the foramen magnum and C1 lamina was perforated with a syringe needle, a PE-10 catheter was advanced 2 mm into the cisterna magna. The catheter was sealed to the dura with tissue glue, and the incision was closed with layered sutures. The outer end of the catheter was connected to an osmotic minipump (Alzet 2004), which was placed under the skin in the neck region. The control group received a continuous infusion of aCSF.

#### Formalin-induced acute inflammatory pain and CFA-induced chronic inflammatory pain.

Rats or mice were briefly anesthetized with isoflurane (2% in O_2_) and received a s.c. injection of CFA (MilliporeSigma, 100 μL for rats and 5 μL for mice) or formalin (MilliporeSigma, 1%, 10 μL) into the plantar surface of the right hind paw. After formalin injection, the animals were immediately placed individually in observation chambers and then monitored for pain behavior (licking and flinching of the injected paw) for 60 minutes. The cumulative duration of pain behavior was counted at 5-minute intervals. The threshold of paw withdrawal responses to von Frey filament stimulation was tested in CFA-injected animals for 14 consecutive days after the CFA injection by an investigator who was blinded to the treatments and genotypes.

#### Hot-plate test.

Mice were gently placed on the hot plate, with the temperature set at 53.0°C ± 0.2°C (IITC Life Sciences). The latency of responses (licking, retraction, or jumping) was recorded. In the absence of a response, the mouse was removed from the hot plate at the 30-second point to avoid tissue injury. The investigator was blinded to the treatments and genotypes.

#### Tail-flick test.

Mice were lightly restrained in small lucite cubicles. One-third of the mouse’s tail was dipped into a water bath with a temperature of 48.0°C. The latency to flick or curl the tail was recorded. A maximum cutoff of 15 seconds was set to avoid tissue injury. The investigator was blinded to the treatments and genotypes.

### Retrograde labeling of spinally projecting neurons in the RVM

Adult rats of either sex were anesthetized with sodium pentobarbital (50 mg/kg, i.p.). A small laminectomy was performed at the fourth thoracic segment, and the dura was punched with a syringe needle. A 1 mm^3^ pledget of spongia gelatinosa soaked with 0.2% DiI (in 100% DMSO, Invitrogen, Thermo Fisher Scientific, D3899) was introduced onto the dorsal surface of the spinal cord. The wound was sutured, and the animal was allowed to recover from anesthesia. The animal was injected with an antibiotic and then placed individually in a cage with free access to food and water. After 10–14 days, the rats were euthanized and perfused for GPER immunofluorescence staining in the RVM region.

### Immunohistochemical analysis

Postfixed brain tissues were transferred to 30% sucrose and incubated at 4°C. OCT compound (Tissue-Tek) was used to embed tissues, and cryostat sectioning was performed for staining. Sections from all animals were immunolabeled using a standardized immunohistochemical procedure. Briefly, free-floating sections were blocked for 1 hour in PBS containing 10% normal goat serum and 1% Triton X-100 and then stained with the following antibodies: rabbit anti-GPER (1:1,000, MBL International, LS-A4272); goat anti–5-HT (1:1,000, Neuromics, GT20079); guinea pig anti-MOR (1:300, Neuromics, GP10106); mouse anti-NeuN (1:1,000, MilliporeSigma, MAB377); mouse anti-KDEL (1:1,000, Abcam, ab12223); mouse anti-TGN46 (1:1,000, Abcam, ab2809); rabbit anti–c-Fos (1:1,000, Cell Signaling Technology, 2250); mouse anti-ERα (prediluted, Abcam, ab51892); mouse anti-ERβ (1:300, Abcam, ab16813); mouse anti-GAD67 (1:1,000, MilliporeSigma, MAB5406); and mouse anti-TPH (1:1,000, MilliporeSigma, T0678). Donkey or goat secondary antibodies (Jackson ImmunoResearch) were used where appropriate. After washing in PBS, sections were mounted onto slides with ProLong Gold Antifade Reagent with DAPI (Invitrogen, Thermo Fisher Scientific) and examined on a conventional fluorescence microscope (Leica DM2500, Leica Microsystems) or confocal microscope (Olympus FV3000, Olympus). In the experiment to compare the rostral-caudal distribution of GPER immunoreactivity, sections of medulla were scanned using a slide scanner (Zeiss Axio Scan.Z1, Carl Zeiss).

### Western blotting

The RVM region (2 mm × 1 mm × 2 mm) was collected under a dissecting microscope and homogenized in lysis buffer containing 20 mM Tris-HCl (pH 8.0), 150 mM NaCl, 1 mM EDTA, 1% NP-40, 1 mM PMSF, a protease inhibitor cocktail (MilliporeSigma), and a phosphatase inhibitor cocktail (Thermo Fisher Scientific) for 1 hour at 4°C. The lysates were centrifuged at 10,000*g* for 20 minutes at 4°C, and the protein concentration in each supernatant was determined using a bicinchoninic acid (BCA) assay (Pierce, Thermo Fisher Scientific). Total protein (20 μg) was electrophoresed on a 10% SDS PAGE gel and transferred onto a PVDF membrane, followed by blocking with 5% nonfat dried milk or 5% BSA. Subsequently, the membranes were incubated with different primary antibodies for 20 hours at 4°C. The primary antibodies rabbit anti-GPER (1:1,000, Abcam, ab39742); rabbit anti-MOR (1:2,000, Immunostar, 24216); and mouse anti–β-actin (1:2000, MilliporeSigma, MAB1501) were used in our study. HRP-conjugated anti–rabbit or anti–mouse secondary antibodies (1:3,000, Bio-Rad) were applied to detect bound primary antibodies. Immunoreactive bands were visualized using enhanced chemiluminescence (Thermo Fisher Scientific). Digital images were captured with an ImageQuant LAS 4000 Mini (GE Healthcare Life Sciences). ImageJ software (NIH) was used to measure the density of specific bands normalized against a β-actin loading control.

### Statistics

Data are presented as the mean ± SEM. Statistical analysis was performed using GraphPad Prism 8 (GraphPad Software). All statistical analyses were 2 tailed. A paired or unpaired Student’s *t* test was used to compare differences between 2 groups. Multiple-group comparisons were performed using 1-way or 2-way ANOVA with Tukey’s or Bonferroni’s post test. Differences were considered statistically significant when a *P* value was less than 0.05.

### Study approval

All animal care and experimental procedures were conducted in compliance with the guiding principles in the Care and Use of Animals and the Animal Management Rule of the Ministry of Public Health, People’s Republic of China (document number 545, 2001) and approved by the Ethics Committee for the Experimental Use of Animals of Shanghai Jiao Tong University School of Medicine (document number SYXK-2013-0050).

## Author contributions

WR, WY, and XS designed the experiments. YJ, PG, LD, YM, XD, JQ, TG, RW, TJ, YZ, DK, YW, SC, SX, DT, PL, MW, and LM performed the experiments and analyzed the data. WR, YJ, and PG wrote the manuscript. DW, CW, GZ, and WY revised the manuscript. WR, YJ, and PG completed the final review and submitted the manuscript. All authors contributed to the article and approved the submitted version. The order of the co–first authors was determined on the basis of their efforts and contributions to the study.

## Supplementary Material

Supplemental data

## Version 1 11/08/2022

In-Press Preview

## Version 2 01/03/2023

Electronic publication
