# Supplementary material for: Molecular identification of bulbospinal ON neurons by GPER, which drives pain and morphine tolerance
Source: J Clin Invest. 2023 Jan 3;133(1):e154588. doi: 10.1172/JCI154588 (PMC9797334; doi:10.1172/JCI154588)
Supplement: Supplemental data [file jci-133-154588-s123.pdf]

Supplemental Material

Molecular identification of bulbospinal ON neurons by GPER which drives pain and morphine tolerance

Authors

Yingfu Jiao[1,2], Po Gao[1,2], Li Dong[1], Xiaowei Ding[1], Youqiang Meng[1,3], Jiahong Qian[1], Ting Gao[1], Ruoxi Wang[2], Tao Jiang[2], Yunchun Zhang[2], Dexu Kong[2], Yi Wu[2], Sihan Chen[2], Saihong Xu[2], Dan Tang[2], Ping Luo[1], Meimei Wu[1], Li Meng[1], Daxiang Wen[2], Changhao Wu[5], Guohua Zhang[1], Xueyin Shi[4#], Weifeng Yu[2#] and Weifang Rong[1,4#].

Affiliations

[1]Department of Anatomy and Physiology, Shanghai Jiao Tong University School of Medicine, Shanghai 200025, China. [2]Department of Anesthesiology, Renji Hospital, Shanghai Jiao Tong University School of Medicine, Shanghai 200127, China. [3]Department of Neurosurgery, Xinhua Hospital, Shanghai Jiao Tong University School of Medicine, Chongming Branch; Shanghai University of Medicine & Health Sciences Affiliated Chongming Hospital, Shanghai 202150, China. [4]Department of Anesthesiology, Xinhua Hospital, Shanghai Jiao Tong University School of Medicine, Shanghai 200092, China. [5]School of Biosciences and Medicine, University of Surrey, Guildford, UK.

Authorship note: YFJ, PG, LD, XWD and YQM contributed equally to this work.

[#]Corresponding authors.

Address correspondence to:

Weifang Rong, Department of Anatomy and Physiology, Shanghai Jiao Tong University School of Medicine, Shanghai, 200025, China. Email: weifangrong@shsmu.edu.cn

Weifeng Yu, Department of Anesthesiology, Renji Hospital, Shanghai Jiao Tong University School of Medicine, Shanghai 200127, China. Email: ywf808@yeah.net.

Xueyin Shi, Department of Anesthesiology, Xinhua Hospital, Shanghai Jiao Tong University School of Medicine, Shanghai 200092, China. Email: shixueyin1128@163.com.

Conflict of interest statement: The authors have declared that no conflict of interest exists.

Supplemental Figures

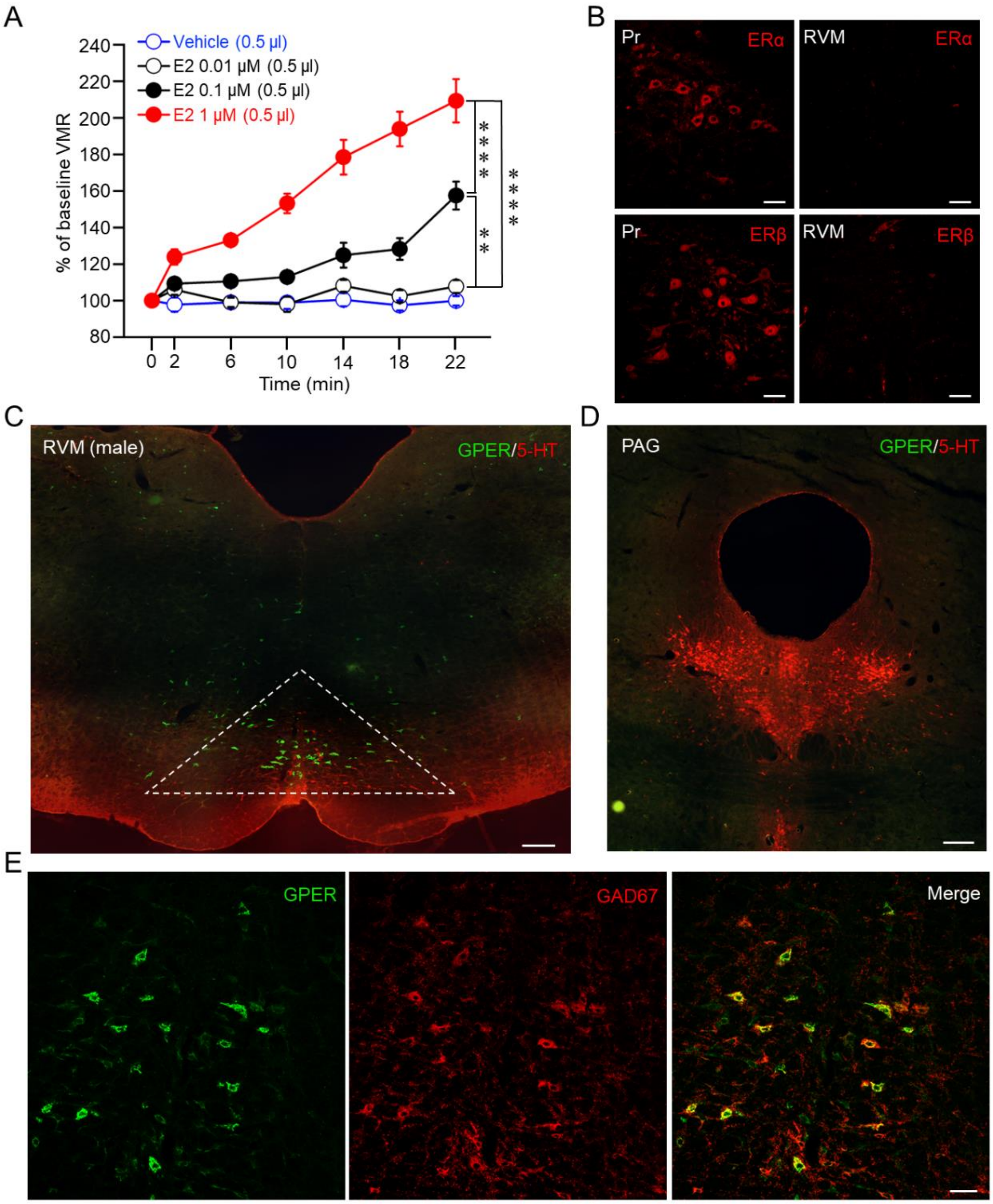

Supplemental Figure 1. GPER is enriched in RVM. (**A**) E2 applied to the RVM resulted the incremental increases in colorectal distension (CRD)-induced visceral motor responses (VMR) in a dose-dependent manner. n=6 female rats for each group. (**B**) Immunofluorescent staining of the medulla oblongata revealed strong ERα and ERβ immunoreactivity in the prepositus nucleus (Pr) but not the RVM. Scale bars: 40 μm. (**C**)

Distinctive GPER immunoreactivity in the RVM of male rats. Scale bar: 200 μm. (**D**) GPER was hardly detectable in PAG and the adjacent serotonergic dorsal raphe nuclei. Scale bar: 200 μm. (**E**) Co-labelling of GPER with GABA synthetase GAD67 in RVM. Scale bar: 50 μm. Data are representative of at least 3 independent experiments. Data are presented as the mean ± SEM. $^{**}P<0.01$ and $^{****}P<0.0001$, by 1-way ANOVA with Tukey post hoc test (**A**).

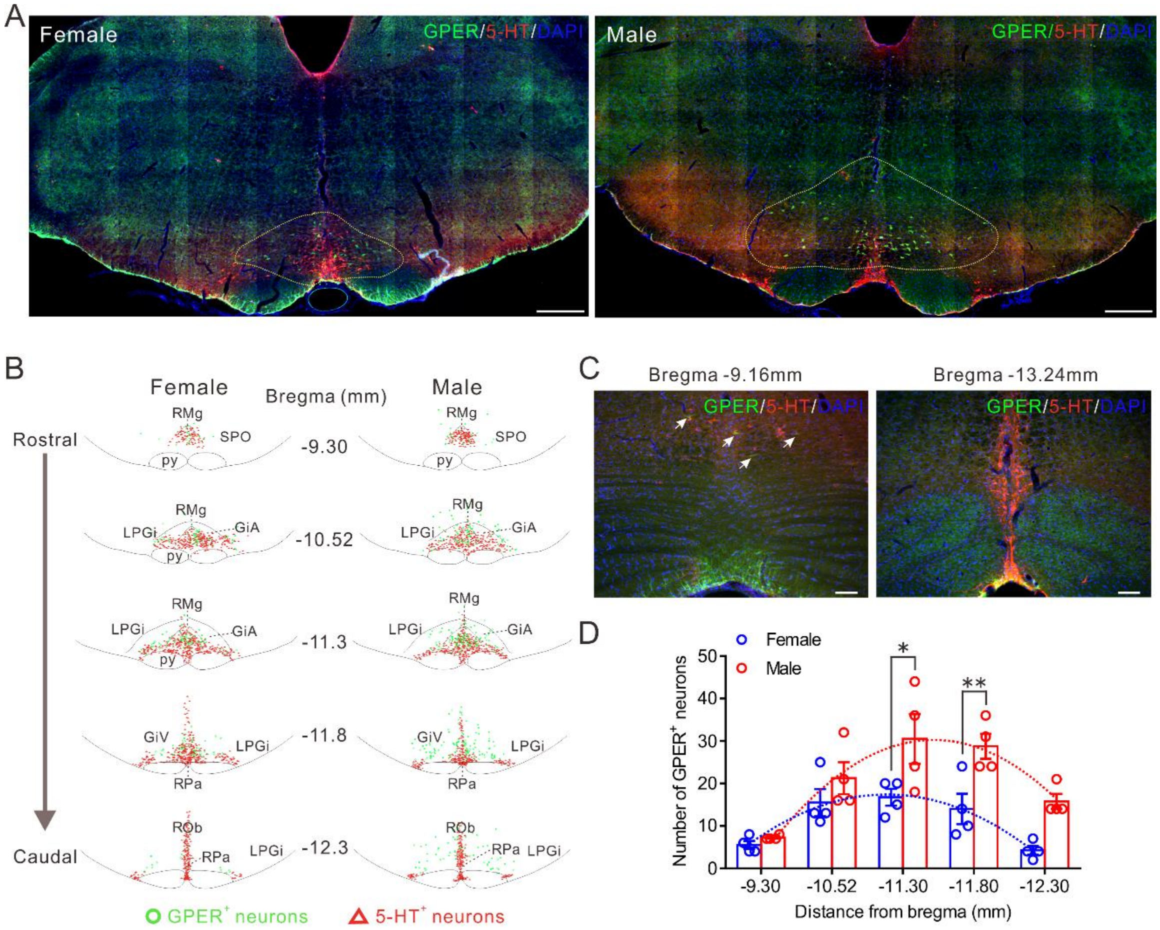

Supplemental Figure 2. The rostral-caudal distribution of GPER$^+$ neurons in the ventral medulla of female and male rats. (**A**) Representative double fluorescence IHC for GPER and 5-HT in a section of medulla from female (left) and male rats (right). Scale bars: 500 μm. (**B**) A plot of the rostral-caudal distribution of GPER$^+$ and 5-HT$^+$ neurons in the medulla in female and male rats (pooled from 4 female and 4 male rats). (**C**) GPER and 5-HT immunofluorescence in the rostral (left) and caudal margin (right) of RVM from female rat. Arrow indicates a few GPER$^+$ neurons are scattered in the rostral margin of RVM. Scale bars: 100 μm. (**D**) Statistical analysis of the rostral-caudal distribution of GPER$^+$ neurons in the RVM of female and male rats. n=4 rats for each group. Data are representative of 4 independent experiments. Data are presented as the mean ± SEM. **P*<0.05 and ***P*<0.01, by 2-way ANOVA followed by Bonferroni post hoc test (**D**).

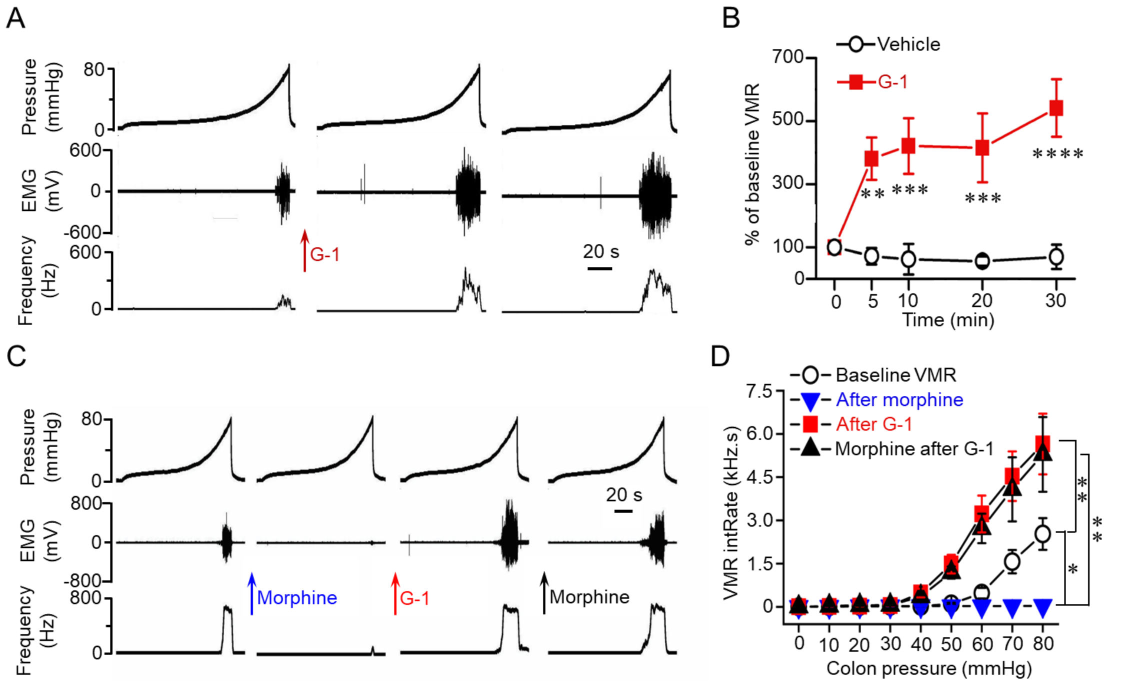

Supplemental Figure 3. Activation of GPER results in colorectal hyperalgesia and attenuation of morphine analgesia. (**A** and **B**) GPER agonist G-1 applied to RVM led to incremental increases in ramp CRD-induced VMR. n=6-7 rats for each group. (**C** and **D**) G-1 applied to RVM negated the analgesic effect of morphine (also microinjected to RVM). n=5 rats. Data are representative of at least 5 independent experiments. Data are presented as the mean ± SEM. *P<0.05, **P<0.01, ***P<0.001 and ****P<0.0001, by 2-way ANOVA followed by Bonferroni post hoc test (**B**) and paired 2-tailed Student's t test (**D**).

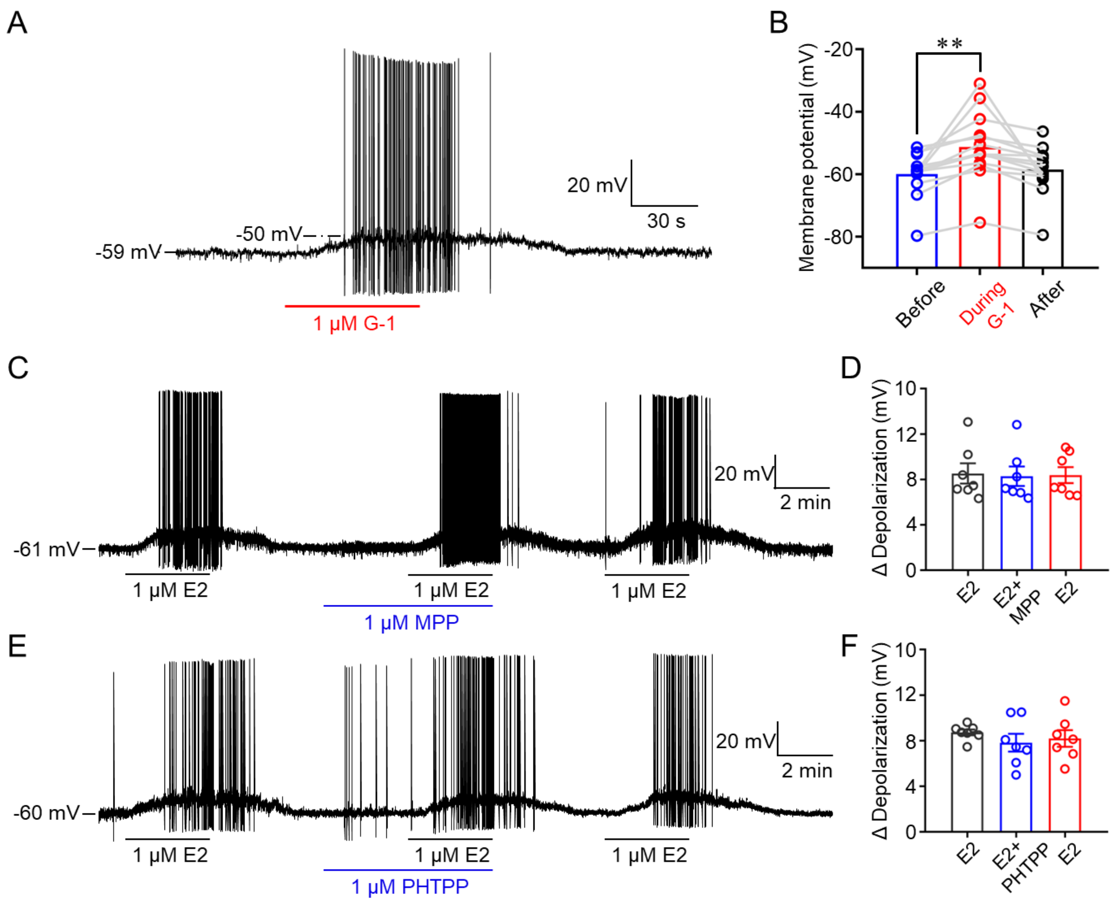

Supplemental Figure 4. GPER mediates E2-induced depolarization of RVM neurons. (**A** and **B**) GPER selective agonist G-1 caused significant depolarization of RVM neurons in medulla slice in vitro. n=14 cells from 3 rats. (**C** and **D**) ERα antagonist MPP was without significant effect on E2-induced depolarization in RVM neurons. n=7 cells from 2 rats. (**E** and **F**) ERβ antagonist PHTPP did not affect E2-induced depolarization of RVM neurons. n=7 cells from 2 rats. Data are representative of at least 2 independent experiments. Data are presented as the mean ± SEM. $^{**}P<0.01$, by paired 2-tailed Student's *t* test (**B**).

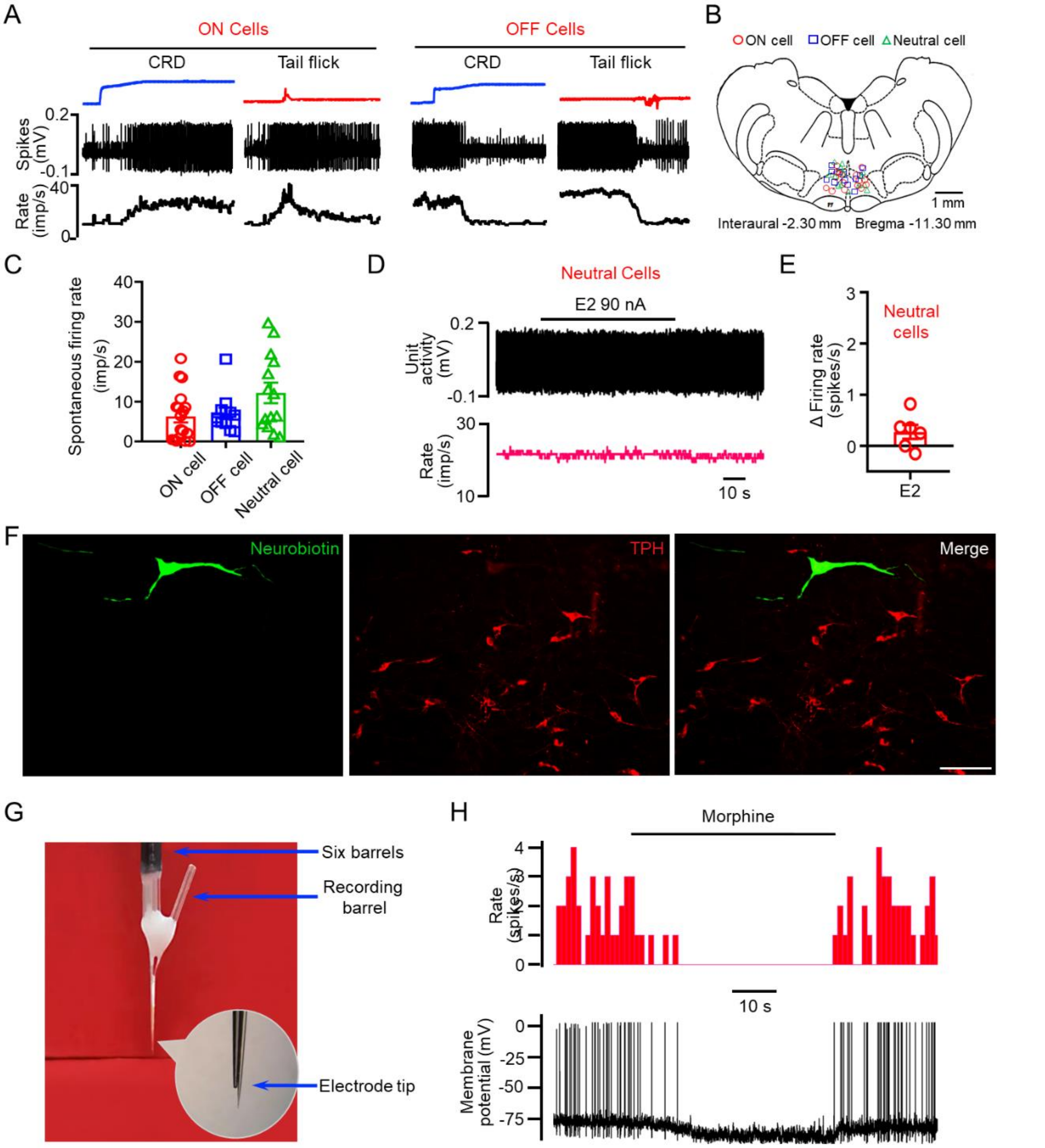

Supplemental Figure 5. Electrophysiological recording and functional identification of ON and OFF neurons in the rat *in vivo.* (**A**) Representative ON (left panels) or OFF cell (right panels) responses to noxious colorectal distention (CRD) and noxious thermal stimulation of the tail (Tail flick). (**B** and **C**) Anatomical position and baseline firing rate of RVM ON, OFF and Neutral cells recorded in our experiments (n=18 for ON cells, n=12 for OFF cells and n=14 for Neutral cells). (**D** and **E**) The firing rate of RVM neutral cells was not affected by iontophoretic application of E2 (n=6). (**F**) Representative IHC images illustrating neurobiotin-labeled RVM ON cell was not immunoreactive to TPH. Scale bar:

50 μm. (**G**) Image of the multi-barrel piggyback electrodes used for intracellular recording and iontophoresis. (**H**) Iontophoretically applied morphine led to hyperpolarization and decreased firing rate of intracellularly-recorded RVM ON cells. Data are representative of at least 3 independent experiments. Data are presented as the mean ± SEM.

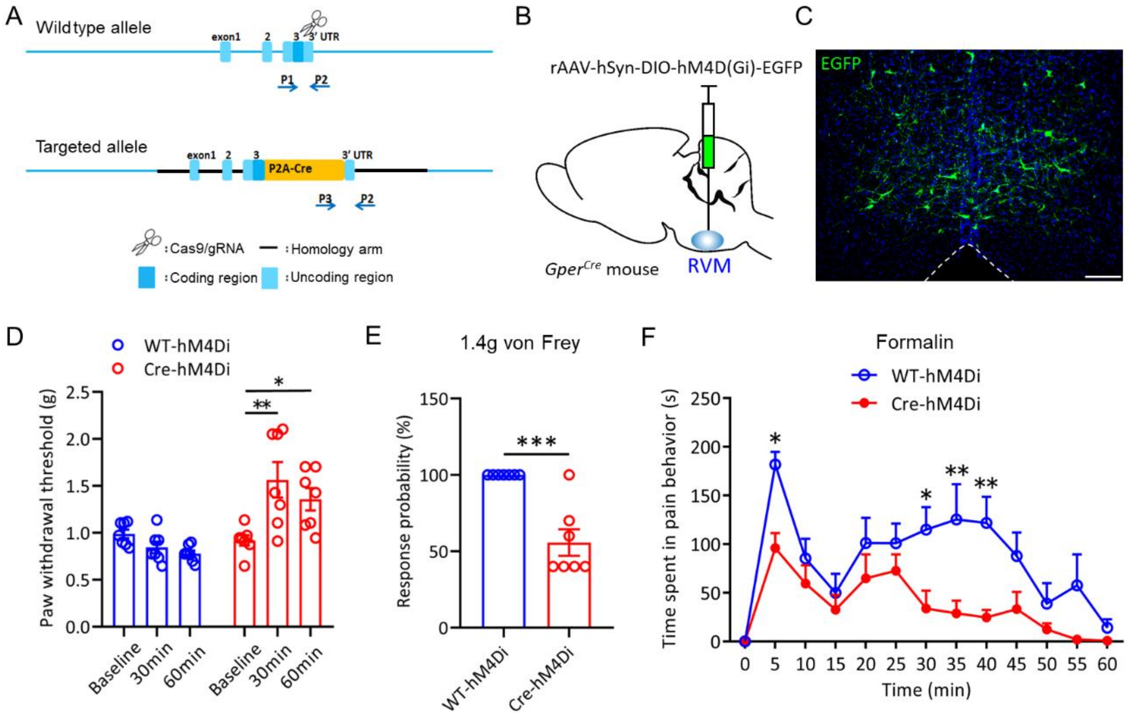

Supplemental Figure 6. Chemogenetic silencing of RVM GPER$^+$ neurons attenuates acute mechanical and inflammatory pain. (**A**) Schematic diagram of *Gper^{Cre}* mice construction strategy. (**B**) Schematic showing the injection of rAAV-hSyn-DIO-hM4D(Gi)-EGFP into the RVM of *Gper^{Cre}* mice. (**C**) Expression of rAAV-hSyn-DIO-hM4D(Gi)-EGFP in the RVM after viral infection. Scale bar: 100 μm. (**D**) Paw withdrawal threshold was significantly increased following clozapine-N-oxide (CNO)-induced chemogenetic silencing of RVM GPER$^+$ neurons. n=7 mice for each group. (E) Frequency of withdrawal responses to repeated probing of hind paw with 1.4g von Frey filaments was decreased following CNO-induced chemogenetic silencing of RVM GPER$^+$ neurons. n=7 mice for each group. (F) Formalin-induced pain behavior was significantly decreased following CNO-induced chemogenetic silencing of RVM GPER$^+$ neurons. n=6 mice for each group. Data are representative of 3 independent experiments. Data are presented as the mean ± SEM. *P<0.05, **P<0.01 and ***P<0.001, by 1-way ANOVA with Tukey post hoc test (**D**), unpaired 2-tailed Student's *t* test (**E**), and 2-way ANOVA followed by Bonferroni post hoc test (**F**).

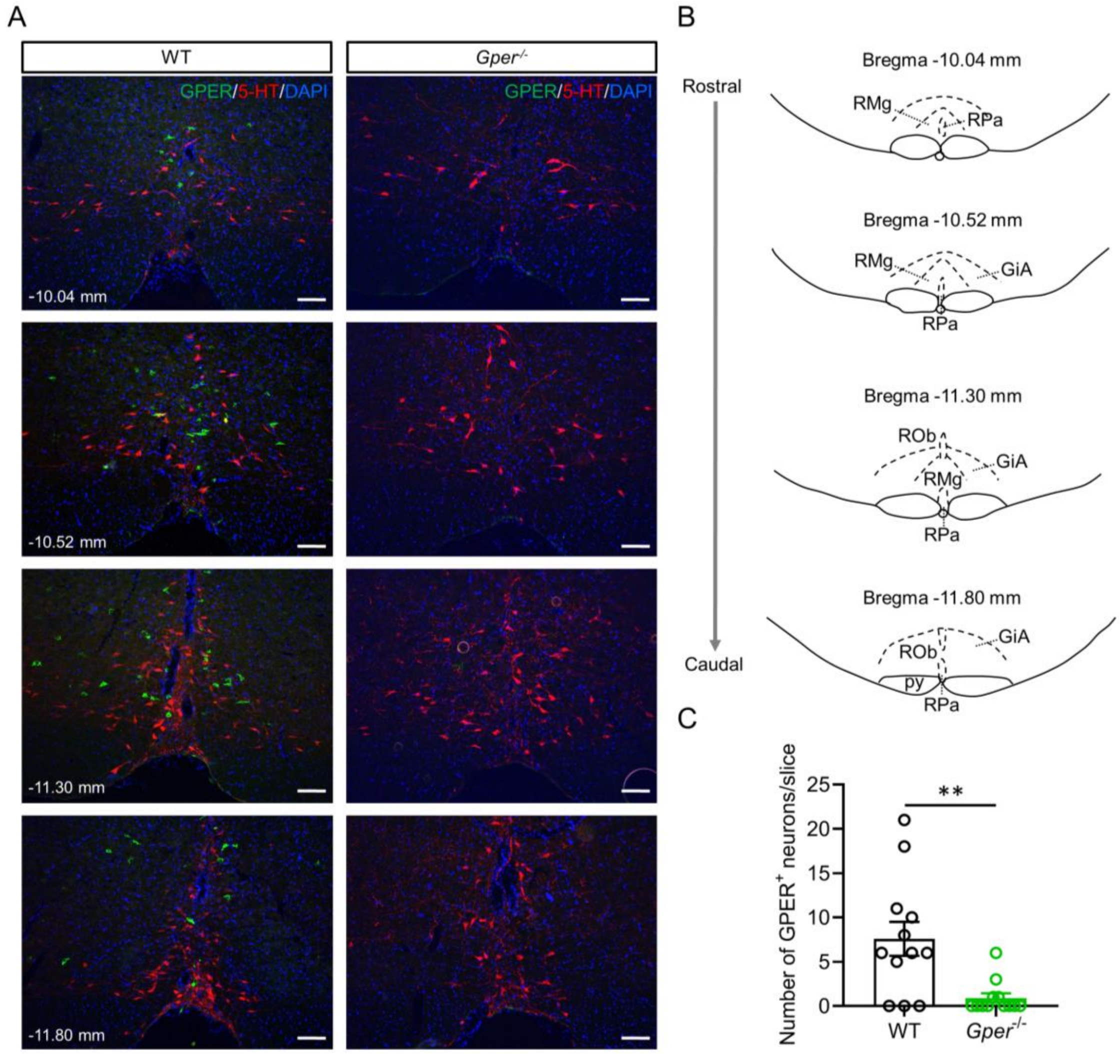

Supplemental Figure 7. Validation of specificity for LS-A4272 by immunofluorescent staining in *Gper*$^{-/-}$ rats. (**A**) Both GPER (green) and 5-HT (red) were abundantly expressed in RVM of WT female rats, and GPER immunoreactivity was exclusively confined to non-serotonergic neurons. The GPER immunoreactivity was markedly reduced in RVM of *Gper*$^{-/-}$ rats, in which only some non-specific green immunofluorescent responses to the GPER primary or secondary antibody could be seen in the middle edge of RVM slice. Scale bars: 100 μm. From top to bottom are representative rostral to caudal RVM sections relative to the bregma, as illustrated in (**B**). (**C**) Statistical analysis of number of GPER^{+} neurons in the RVM of WT and *Gper*$^{-/-}$ female rats. n=12 slices from 2 female rat for each group. Data are representative of 2 independent experiments. Data are presented as the mean ± SEM. ***P*<0.01, by unpaired 2-tailed Student's *t* test (**C**).

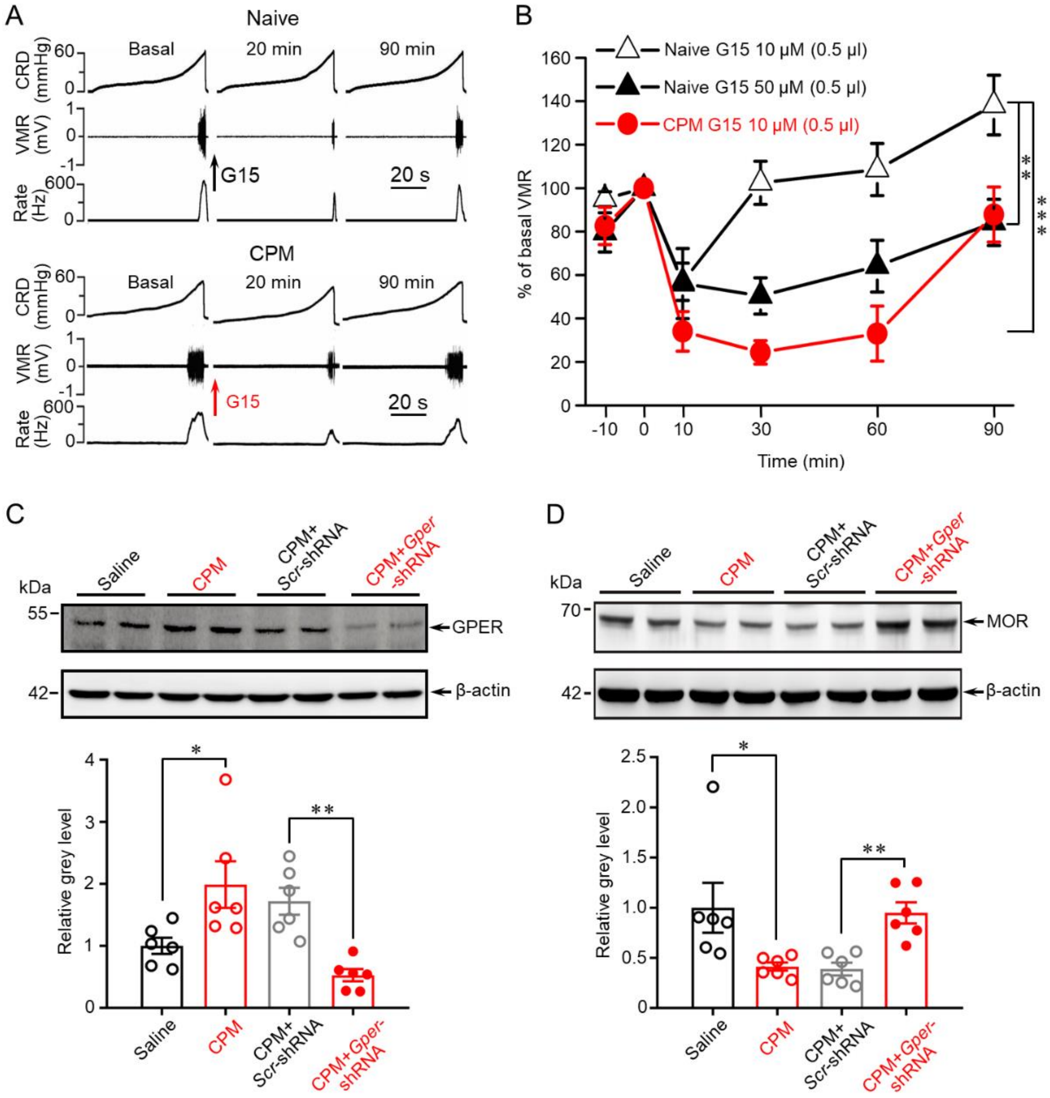

Supplemental Figure 8. GPER signaling contributes to CPM-induced colorectal hypersensitivity. (**A** and **B**) The effect of G15 microinjected to RVM on ramp CRD-induced visceral motor responses (VMR) in Naive or CPM-treated rats. n=6 rats for each group. (**C**) GPER expression was increased in CPM model, while shRNA treatment knock down GPER in the RVM of CPM-treated female rats. n=6 rats for each group. (**D**) MOR expression was decreased in CPM model, while *Gper* knockdown shRNA treatment reversed the down-regulation of MOR in RVM of CPM-treated female rats. n=6 rats for each group. Data are representative of at least 2 independent experiments. Data are presented as the mean ± SEM. *P<0.05, **P<0.01 and ***P<0.001, by 1-way ANOVA with Tukey post hoc test (**B**-**D**).

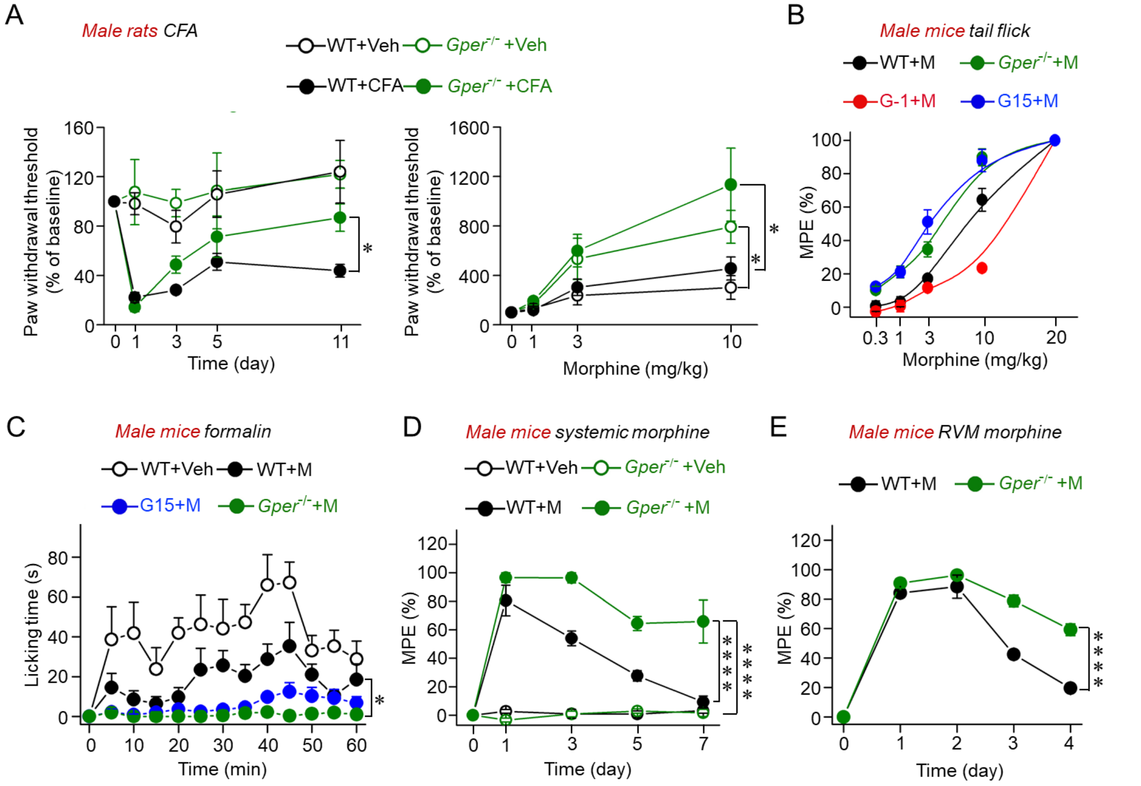

Supplemental Figure 9. GPER signaling modulates morphine analgesia and tolerance in male rats and mice. (**A**) *Gper*$^{-/-}$ male rats displayed decreased hyperalgesia (left panel) and higher sensitivity to morphine analgesia compared with the WT male rats following intraplantar injection of CFA (right panel). n=5-6 rats. (**B**) In the tail flick test, either pharmacological blockade of GPER with G15 or genetic ablation of GPER (*Gper*$^{-/-}$) led to a leftward shift of the dose-response curve of morphine analgesia in male rats. n=5-7 rats for each group. (**C**) In the formalin test, either pharmacological blockade of GPER with G15 or genetic ablation of GPER (*Gper*$^{-/-}$) led to enhanced morphine analgesia in male mice. n=5-6 mice. (**D**) Development of systemic morphine tolerance was slowed in *Gper*$^{-/-}$ male mice compared with the wild type male mice. n=5 mice for each group. (**E**) Development of tolerance to locally-injected (RVM) morphine was slowed in *Gper*$^{-/-}$ male mice. n=6 mice for each group. Data are representative of at least 3 independent experiments. Data are presented as the mean ± SEM. *P<0.05 and ****P<0.0001, by 1-way ANOVA with Tukey post hoc test (**A-D**) and unpaired 2-tailed Student's *t* test (**E**).

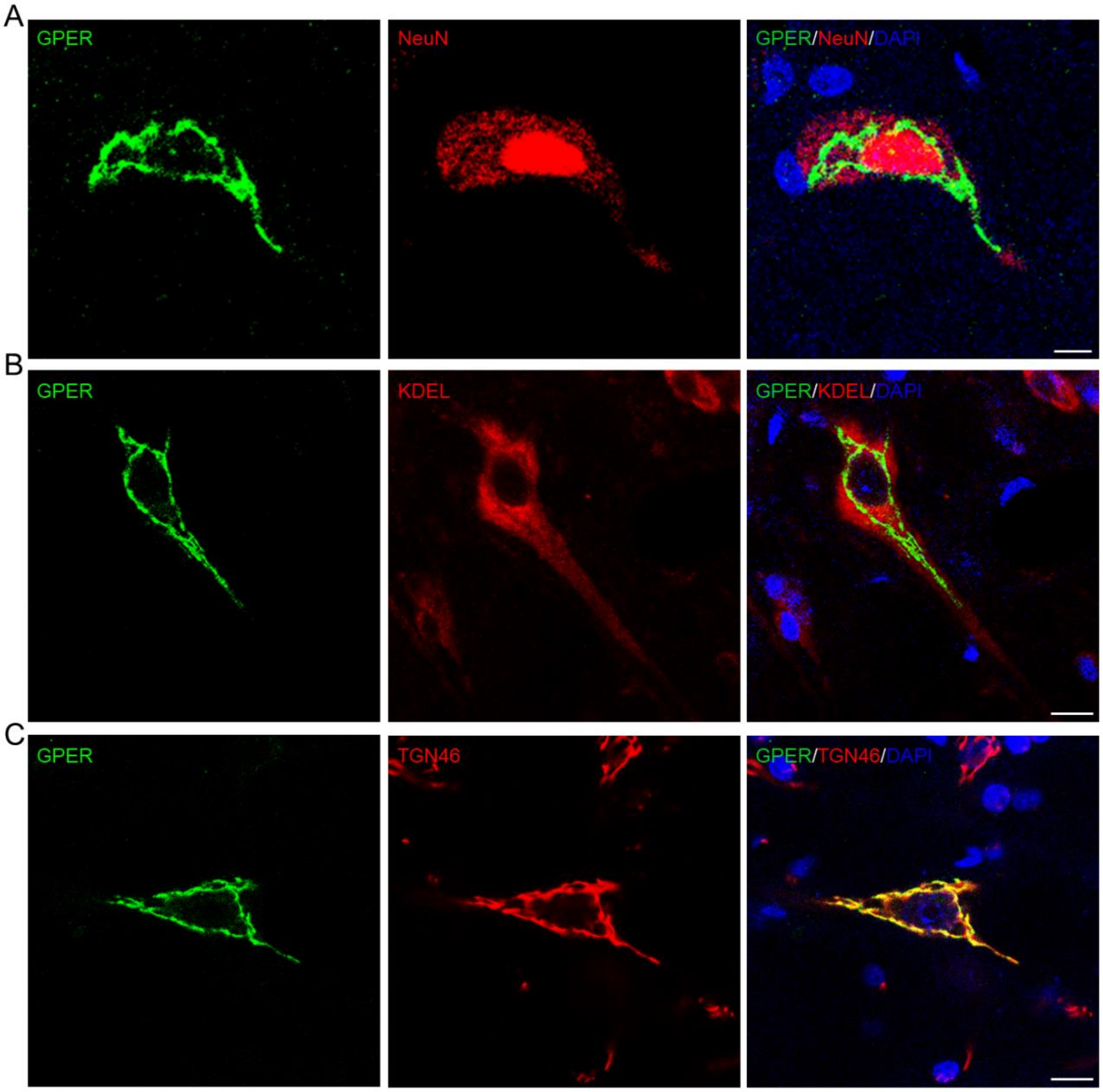

Supplemental Figure 10. GPER seems to be distinctively located on the golgi apparatus. (**A**) Double staining for GPER and NeuN (a neuronal marker) showing intracellular localization of GPER immunoreactivity in RVM neurons. Scale bar: 5 μm. (**B**) Double staining for GPER and KDEL (which stains endoplasmic reticulum) excluded localization of GPER in ER. Scale bar: 10 μm. (**C**) Double staining for GPER and TGN46 (a marker of Golgi apparatus) showing localization of GPER in Golgi apparatus. Scale bar: 10 μm. Data are representative of 3 independent experiments.
